# ERICA: intake of macro and micronutrients of Brazilian adolescents

**DOI:** 10.1590/S01518-8787.2016050006698

**Published:** 2016-02-02

**Authors:** Amanda de Moura Souza, Laura Augusta Barufaldi, Gabriela de Azevedo Abreu, Denise Tavares Giannini, Cecília Lacroix de Oliveira, Marize Melo dos Santos, Vanessa Sá Leal, Francisco de Assis Guedes Vasconcelos

**Affiliations:** I Instituto de Estudos em Saúde Coletiva. Universidade Federal do Rio de Janeiro. Rio de Janeiro, RJ, Brasil; II Departamento de Vigilância de Doenças e Agravos Não Transmissíveis e Promoção da Saúde. Secretaria de Vigilância em Saúde. Ministério da Saúde. Brasília, DF, Brasil; III Programa de Pós-Graduação em Saúde Coletiva. Instituto de Medicina Social. Universidade do Estado do Rio de Janeiro. Rio de Janeiro, RJ, Brasil; IV Divisão de Nutrição. Hospital Universitário Pedro Ernesto. Universidade do Estado do Rio de Janeiro. Rio de Janeiro, RJ, Brasil; V Departamento de Nutrição. Universidade do Estado do Rio de Janeiro. Rio de Janeiro, RJ, Brasil; VI Departamento de Nutrição. Núcleo de Estudos em Saúde Pública. Universidade Federal do Piauí. Teresina, PI, Brasil; VII Núcleo de Nutrição. Centro Acadêmico de Vitória. Universidade Federal de Pernambuco. Vitória de Santo Antão, PE, Brasil; VIII Departamento de Nutrição. Centro de Ciências da Saúde. Universidade Federal de Santa Catarina. Florianópolis, SC, Brasil

**Keywords:** Adolescent, Food Consumption, Micronutrients, Macronutrients, Diet Surveys, Cross-Sectional Studies, Nutrition Surveys

## Abstract

**OBJECTIVE:**

To describe food and macronutrient intake profile and estimate the prevalence of inadequate micronutrient intake of Brazilian adolescents.

**METHODS:**

Data from 71,791 adolescents aged from 12 to 17 years were evaluated in the 2013-2014 Brazilian Study of Cardiovascular Risks in Adolescents (ERICA). Food intake was estimated using 24-hour dietary recall (24-HDR). A second 24-HDR was collected in a subsample of the adolescents to estimate within-person variability and calculate the usual individual intake. The prevalence of food/food group intake reported by the adolescents was also estimated. For sodium, the prevalence of inadequate intake was estimated based on the Tolerable Upper Intake Level (UL). The Estimated Average Requirement (EAR) method used as cutoff was applied to estimate the prevalence of inadequate nutrient intake. All the analyses were stratified according to sex, age group and Brazilian macro-regions. All statistical analyses accounted for the sample weight and the complex sampling design.

**RESULTS:**

Rice, beans and other legume, juice and fruit drinks, breads and meat were the most consumed foods among the adolescents. The average energy intake ranged from 2,036 kcal (girls aged from 12 to 13 years) to 2,582 kcal (boy aged from14 to 17 years). Saturated fat and free sugar intake were above the maximum limit recommended (< 10.0%). Vitamins A and E, and calcium were the micronutrients with the highest prevalence of inadequate intake (> 50.0%). Sodium intake was above the UL for more than 80.0% of the adolescents.

**CONCLUSIONS:**

The diets of Brazilian adolescents were characterized by the intake of traditional Brazilian food, such as rice and beans, as well as by high intake of sugar through sweetened beverages and processed foods. This food pattern was associated with an excessive intake of sodium, saturated fatty acids and free sugar.

## INTRODUCTION

The leading causes of death in all regions of Brazil are chronic non-communicable diseases (NCD)^19^. Being overweight and obese, which are important risk factors for these diseases, are showing increasing levels of prevalence worldwide and can affect all stages of life, including childhood and adolescence^9^.

Increased consumption of ultra-processed foods, which are high in fat, sugar and salt, and decreased consumption of legume, vegetables and fruits^20^, which are associated with lower daily energy expenditure, can explain the growing trends for being overweight and obese^2^ ^,^ ^a^, as well as metabolic changes in the child and adolescent population. These aspects also contribute to nutritional deficiencies that are characteristic during this stage of life, such as those regarding iron, zinc, calcium, phosphorus and vitamins A, C, and E^21^.

Adolescence is a time of intense body modification^b^; improper eating habits during this period are associated with increased risk of obesity and other NCD^8^, so monitoring the food consumed by Brazilian adolescents is an important factor for implementing and evaluating intervention strategies. Based on the aforementioned, the objective of this study was to describe the profile of food and macronutrient consumption as well as to estimate the prevalence of inadequate intake of these micronutrients of Brazilian adolescents.

## METHODS

The Brazilian Study of Cardiovascular Risks in Adolescents (ERICA), performed from 2013 to 2014, was used as a data source. ERICA is a national school-based survey whose goal was to evaluate the prevalence of cardiovascular risk factors and metabolic syndrome in adolescents aged between 12 and 17 years who attended public and private schools in 124 cities. Detailed information regarding the sampling procedure and data collection have already been published^3^ ^,^ ^23^. In brief, ERICA adopted a three stage cluster sampling plan. During the first stage, the schools were selected with probability proportional to size, which had been previously stratified into 32 geographical strata (27 state capitals and five sets with the other municipalities from each macro-region). The second stage saw three combinations of class sessions (morning and afternoon) and year (one of the last three years of elementary school or one of the first three years of high school) being selected. One class for each of the previously described combinations was selected in the third stage^15^. Adolescents not within the 12 to 17 years age group were excluded, as well as pregnant adolescents and those with some degree of disability that made anthropometric evaluation or completing the questionnaire impossible.

Of the 102,327 adolescents who were eligible to participate in ERICA, 73,160 completed their 24-hour dietary recall (24-HDR) and 75,589 filled in a questionnaire (around 100 questions divided into 11 blocks, covering sociodemographic, health and lifestyle aspects) on an electronic data collector (personal digital assistant – PDA). ERICA participants were grouped into subsets according to the parts of the study about which they possessed information, which was done so that the sampling weights were calculated for each of the defined subsets. Therefore, 71,971 adolescents who had completed data for the PDA and 24-HDR subset were evaluated in this study. The non-response rate for this subset was 29.7%. In a subsample of two adolescents per class (around 7.0% of the sample), a second round of 24-HDR were collected to estimate intrapersonal variance, which made it possible to calculate the normal dietary intake of these adolescents.

Food intake was estimated by applying the 24-HDR. The adolescents were interviewed by trained field researchers, who used a specific software to enter food consumption data, by directly recording the information on netbooks. The multiple-pass method was used as interview technique^5^ *,* which consists of an overseen five-stage interview, with the aim of reducing under-report of food consumption. The software used in this study contained a list of items included in the food and beverage purchase database from the 2002-2003 *Pesquisa de Orçamentos Familiares* (POF – Brazilian Household Budget Survey), which was performed by the Brazilian Institute of Geography and Statistics (IBGE)^c^. The food items that were not contained in the database were added by the interviewers.

The intake of energy and nutrients was estimated based on the *Tabela de Composição Nutricional dos Alimentos Consumidos no Brasil* (Brazilian Food Composition Table)^d^ and the *Tabela de Medidas Referidas para os Alimentos Consumidos no Brasil* (Brazilian Portion Size Table)^e^.

Data regarding nutrient intake did not include the consumption of supplements or medicines. To analyze the intake of energy and nutrients, added soy oil was considered in all forms of cooked and braised meat and vegetable preparations. Habitual consumption of sugar and sweetener was evaluated based on the following question: “use frequently; with the following response options: sugar, sweetener, sugar and sweetener, do not use. An addition of 10 g of sugar for every 100 ml of fruit juice, coffee, coffee with milk, tea and herbal tea was standard when adolescents reported habitually consuming sugar; and an addition of 5 g of sugar for every 100 ml of these drinks when the consumption of sugar and sweetener was reported as normal. The 1,626 food items that were available in the list of foods of the software used for data collection during ERICA were categorized into 35 groups with similar macronutrient profiles (Table 1). One single day of consumption provides good estimates for the population’s average intake of nutrients and foods^22^; therefore, prevalence estimates for food consumption, population averages for energy intake and the percentage contribution of macronutrients were calculated based on one 24-HDR. Only 20 of the most consumed foods were presented.

**Table 1:** Categorization of foods mentioned by the participants of ERICA according to a similar macronutrient profile. ERICA, Brazil, 2013-2014.

Food groups	Description
Rice	Rice, rice with vegetables, sushi and other rice-based preparations
Corn	Corn, cornmeal, polenta and other corn-based preparations
Beans and other legumes	Beans, soy meat and other types of beans
Vegetables	Leafy greens and legumes
Tubers	Potatoes, not including industrialized forms (chips), cassava, yams and other tubers
Fruits	Fruits and fruit salads
Oilseeds	Peanuts, cashews, almonds and others
Breakfast cereals	Oats, cereal, Granola bar and other cereals
Pasta	Pasta, ravioli, lasagna and other pasta-based preparations
Soups	Soups and broths
Breads	White and integral and toasted breads
Cakes and pastries	Cakes and pastries in general
Sweet biscuits	Sweet and filled biscuits
Savory biscuits	Savory biscuits and chips (potato or corn)
Meat	Meat, meat-based preparations and other meats
Pork	Pork and pork-based preparations
Chicken	Chicken, chicken-based preparations and other fowl
Fish	Fish and fish-based preparations
Processed meats	Ham, salami, mortadella, sausage, sausage and other processed meats
Eggs	Eggs and egg-based preparations
Milk	Whole and skimmed milk
Flavored dairy drinks	Dairy drinks sweetened with artificial or natural flavorings, and fermented milk
Soy-based beverages	Soy milk and soy-based beverages
Juices and fruit drinks	Natural and processed fruit juices
Soft drinks	Regular soft drinks
Low sugar or light fat soft drinks	Diet and light soft drinks
Coffee	Coffee, cappuccino, latte and other coffee-based drinks
Tea	Teas
Alcoholic beverages	Wine, beer and others
Cheeses and other dairy products	Cheeses and yogurts
Sweets and desserts	Sweets, fruit-based desserts, chocolate and other candies
Sugar, honey and jellies	Sugar, honey and jellies
Diet or light sweets and desserts	Diet or light sweets, desserts, cakes, pastries and cookies
Oils and fats	Vegetable oils, olive oil, butter, margarine, sauces and condiments
Pizza	Pizzas and calzones
Fried and baked snacks	Savory chicken pastry, pie, cheese-bread and other savory snacks
Sandwiches	Hamburgers and other sandwiches

The percentiles of intake distributions and prevalence of inadequate micro-nutrient consumption (calcium, phosphorus, iron, sodium, zinc, vitamins A, C, E and B12) were estimated based on the 24-HDR data, which were corrected for within-person variability in accordance with the method proposed by the National Cancer Institute (NCI)^7^. This method consists of a two-part nonlinear mixed model: the first being based on a random effects logistic regression model to estimate consumption probability; the second estimating the amount consumed through random effect linear regression models, which were applied after data transformed to normality. Brazil’s five macro-regions and the school’s locational status (urban or rural) were considered as covariates in all models used.

The inadequacy prevalences were estimated as the proportion of adolescents who had a micronutrient intake below the estimated average requirement^9^ ^-^ ^16^, using the Estimated Average Requirement (EAR) method as a cut-off point, as recommended by the Institute of Medicine (IOM)^11^. Calculating the prevalence of inadequacy considered the sample weight and the complexity of the sample design, which involved using the Fay-modified Balanced Repeated Replication (Fay-BRR) technique^1^ ^,^ ^6^.

A manually determined probabilistic approach was used to estimate the inadequacy of iron, since the distribution curve of iron necessity is considered asymmetric among women of childbearing age, not taking into account the assumptions required so that the EAR can be used^12^. For each percentile (1, 5, 10, 15, 25, 40, 50, 75, 85, 90, 95, 99) of the distribution of normal iron intakes, the probability of inadequacy was estimated according to sex and age group, based on the recommendations set out by the IOM^12^. The prevalence of inadequacy is the sum of the percentage of adolescents with inadequacy in each percentile.

Regarding sodium intake, values above the Tolerable Upper Intake Level were considered as inadequate, which allowed the proportion of adolescents at risk of adverse health effects to be estimated^10^.

Age was categorized into two groups due to the different recommended micronutrient intake levels for each sex and ages. The analyses were stratified according to sex, age group (12 to 13 years and 14 to 17 years) and macro-regions. All estimates were calculated using the SAS (Statistical Analysis System) software, version 9.3, which took factors regarding the expansion and complexity of the sample design into account.

ERICA was approved by the Research Ethics Committees of the Institute of Collective Health Studies at the Universidade Federal do Rio de Janeiro and those from each State including the Federal District. All participants signed an assent form.

## RESULTS

**Figure 1:**
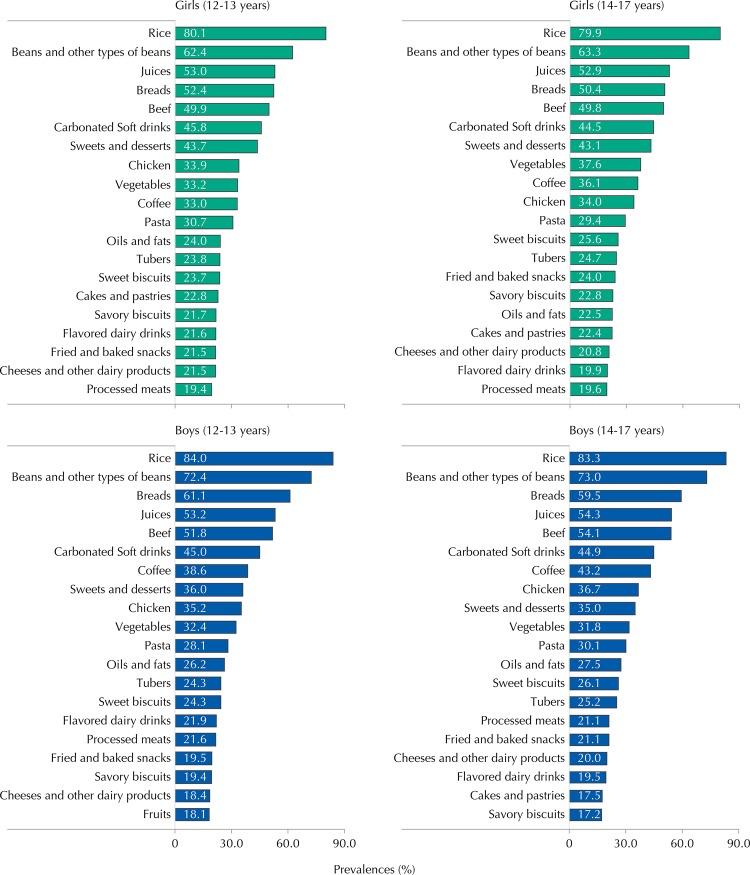
Prevalence of the 20 most consumed foods among Brazilian adolescents according to sex and age group. ERICA, 2013-2014.

Foods that were most prevalently consumed among adolescents in the two age groups were rice (82.0%), beans (68.0%), juices and fruit drinks (56.0%), bread (53.0%) and meat (52.0%) (Figure 1). We observed high prevalence of ultra-processed food consumption, which include carbonated soft drinks, fried and baked snacks, sweet and savory biscuits, with carbonated soft drinks being the sixth most mentioned food (45.0%). The prevalence of fruit consumption was low, with this food group only being listed as most consumed among boys aged between 12 and 13 years (18.0%).

**Figure 2:**
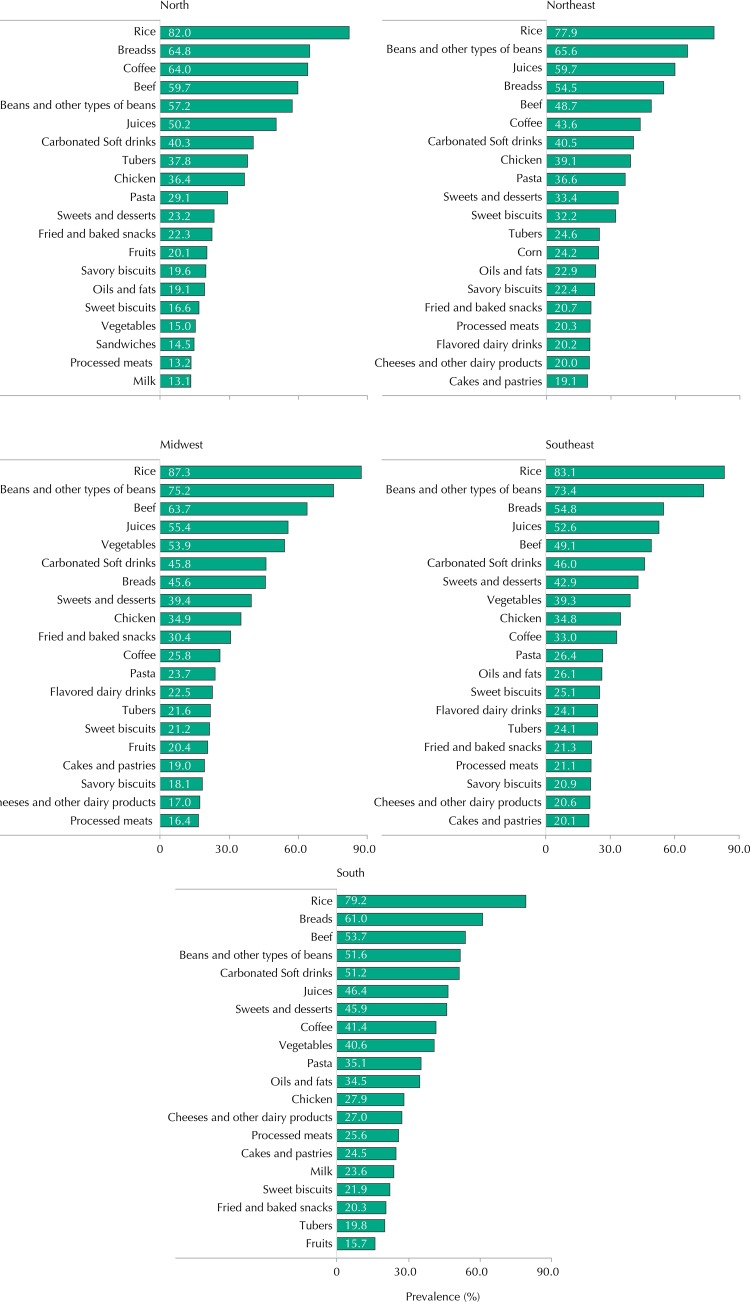
Prevalence of the 20 most consumed foods among Brazilian adolescents according macro-region. ERICA, 2013-2014.

The stratified analysis according to macro-regions showed important differences regarding the rate of consumption of some food items (Figure 2). Coffee (64.0%) was only among the five most consumed foods in the North region. Beans were the second most consumed food in the Southeast, Midwest and Northeast regions. The South region showed the highest prevalence for carbonated soft drink consumption (51.0%). Vegetables (54.0%) only figured among the five most consumed foods in the Midwest region.

The average energy intake of adolescents varied from 2,036 kcal among girls aged between 12 and 13 years to 2,582 kcal among boys aged between 14 and 17 years (Table 2). Total energy intake according to age did not differ for females. For males, energy intake was higher among boys aged between 14 and 17 years, when compared to boys between 12 and 13 years (2,582 kcal *versus* 2,281 kcal). Energy intake averages were similar among the macro-regions.

**Table 2:** Mean and 95%CI of energy consumption and the percentage of the total caloric intake of macronutrients according to sex and age group, both for Brazil and its macro-regions. ERICA, 2013-2014.

Variable	Girls				Boys			
	
	12-13		14-17		12-13		14-17	
			
	Mean	95%CI	Mean	95%CI	Mean	95%CI	Mean	95%CI
Energy (kcal)								
Brazil	2,036	1,981-2,090	2,124	2,086-2,161	2,281	2,192-2,370	2,582	2,511-2,653
North	2,121	1,990-2,253	2,155	2,103-2,206	2,261	2,135-2,387	2,573	2,499-2,648
Northeast	2,100	1,924-2,277	2,257	2,188-2,326	2,502	2,349-2,657	2,668	2,568-2,767
Midwest	2,068	1,988-2,148	2,112	2,052-2,172	2,394	2,273-2,516	2,652	2,564-2,741
Southeast	2,018	1,961-2,076	2,085	2,024-2,144	2,213	2,062-2,364	2,564	2,433-2,696
South	1,913	1,728-2,098	2,033	1,945-2,121	2,121	1,980-2,261	2,464	2,393-2,536
Carbohydrates (%)								
Brazil	54.0	53.4-54.3	53.7	53.4-54.1	53.7	52.9-53.9	53.7	52.8-54.0
North	54.8	53.9-55.7	54.8	53.5-54.4	54.7	53.6-55.9	54.8	54.2-55.5
Northeast	54.6	53.5-55.6	54.0	53.5-54.6	54.4	53.5-55.2	54.6	53.7-55.4
Midwest	53.2	51.9-54.4	53.0	52.2-53.8	52.1	50.9-53.3	51.5	50.7-52.3
Southeast	53.5	52.8-54.1	53.5	52.9-54.1	53.1	52.5-53.8	52.9	51.9-53.9
South	54.5	53.5-55.5	54.3	52.7-55.8	52.2	49.5-54.9	54.8	52.6-55.0
Protein (%)								
Brazil	15.4	15.1-15.7	15.4	15.2-15.6	16.2	15.9-16.4	16.3	16.0-6.6
North	15.6	15.1-16.1	15.6	15.4-15.9	16.3	15.8-16.9	16.2	15.9-16.5
Northeast	15.1	14.7-15.4	15.1	14.7-15.6	15.9	15.2-16.6	15.7	15.5-16.0
Midwest	15.9	15.4-16.5	15.8	15.5-16.1	16.7	16.0-17.4	17.1	16.7-17.5
Southeast	15.6	15.0-16.1	15.6	15.3-15.9	16.2	15.9-16.6	16.5	16.0-17.1
South	14.8	14.0-15.5	14.7	14.1-15.3	16.2	15.3-17.0	15.8	15.4-16.2
Total lipids (%)								
Brazil	30.9	30.5-31.3	31.1	30.7-31.5	30.6	30.2-31.0	30.2	29.8-30.6
North	29.5	29.0-30.0	30.6	30.0-31.2	28.8	28.1-29.5	28.7	28.2-29.1
Northeast	30.6	29.7-31.4	31.0	30.4-31.6	29.8	29.1-30.5	29.6	29.7-30.5
Midwest	31.3	30.4-32.1	31.5	31.0-32.0	31.4	30.6-32.2	31.5	31.0-32.0
Southeast	31.3	30.6-32.0	31.2	30.5-31.8	30.9	30.3-31.5	30.4	29.9-31.0
South	31.1	30.3-31.9	31.3	30.3-32.3	31.7	29.9-33.6	30.4	29.4-31.4
Saturated fatty acids (%)								
Brazil	11.3	11.1-11.7	11.5	11.3-11.7	10.9	10.6-11.1	10.8	10.7-11.0
North	10.5	10.1-10.8	11.2	10.8-11.5	10.3	10.0-10.7	10.2	9.9-10.5
Northeast	11.3	10.8-11.7	11.5	11.1-11.9	10.8	10.3-11.3	10.8	10.3-11.3
Midwest	11.5	10.9-12.2	11.4	11.1-11.7	11.1	10.7-11.5	10.9	10.7-11.2
Southeast	11.6	11.1-12.0	11.5	11.2-11.9	10.9	10.6-11.2	10.9	10.7-11.1
South	11.2	10.7-11.6	11.4	11.0-11.9	11.0	10.3-11.8	10.9	10.5-11.3
Monounsaturated fatty acids (%)								
Brazil	10.5	10.3-11.7	10.6	10.4-10.7	10.3	10.0-10.5	10.2	10.0-10.6
North	10.3	10.0-10.6	10.3	10.2-10.5	10.0	9.7-10.2	9.9	9.7-10.0
Northeast	10.8	10.6-11.0	11.0	10.8-11.1	10.4	10.2-10.6	10.4	10.3-10.5
Midwest	10.9	10.5-11.2	10.9	10.7-11.1	10.6	10.4-10.9	10.8	10.6-11.0
Southeast	10.3	10.1-10.5	10.4	10.3-10.6	10.3	10.8-10.4	10.1	10.0-10.3
South	10.9	10.5-11.2	10.7	10.5-10.9	10.6	10.3-10.9	10.7	10.5-10.9
Polyunsaturated fatty acids (%)								
Brazil	6.2	6.1-6.3	6.1	6.0-6.2	6.2	5.9-6.5	5.9	5.8-6.0
North	6.0	5.7-6.3	5.9	5.7-6.0	5.4	5.1-5.7	5.4	5.3-5.6
Northeast	5.7	5.4-5.9	5.8	5.6-6.0	5.5	5.3-5.7	5.4	5.3-5.5
Midwest	5.9	5.7-6.2	6.2	6.0-6.3	6.1	5.8-6.4	6.1	6.0-6.3
Southeast	6.4	6.2-6.6	6.2	6.0-6.4	6.7	6.1-7.2	6.1	5.9-6.3
South	6.5	6.1-6.9	6.4	6.1-6.7	6.6	6.3-6.9	6.2	5.9-6.5
Total trans-fatty acids (%)								
Brazil	1.0	0.9-1.0	1.0	0.9-1.0	1.0	0.9-1.0	1.0	0.9-1.0
North	1.1	1.0-1.2	1.1	1.0-1.2	1.1	1.0-1.2	1.1	1.0-1.2
Northeast	1.0	0.9-1.1	1.0	0.9-1.0	1.0	0.9-1.2	1.0	1.0-1.2
Midwest	0.9	0.9-0.9	0.9	0.9-1.0	1.0	1.0-1.2	1.0	0.9-1.0
Southeast	0.9	0.8-1.0	0.9	0.9-1.0	1.0	0.8-1.1	1.0	0.9-1.1
South	1.3	1.1-1.5	1.2	1.1-1.2	1.3	1.2-1.4	1.3	1.2-1.4
Total sugar (%)								
Brazil	24.7	24.1-25.3	24.7	24.2-25.1	21.8	21.0-22.5	21.9	21.4-22.4
North	21.3	19.6-22.9	21.5	20.9-22.1	19.2	18.0-20.4	19.1	18.4-19.7
Northeast	24.1	23.3-24.9	24.7	23.7-25.6	22.3	21.3-23.4	22.0	21.3-22.8
Midwest	26.5	25.5-27.6	25.5	24.9-26.1	22.8	21.7-23.9	22.4	21.6-23.2
Southeast	24.7	23.7-25.7	24.7	24.1-25.3	21.6	20.3-22.9	21.8	21.0-22.7
South	27.0	26.0-28.1	26.3	24.8-27.7	22.6	20.6-24.5	23.9	23.2-24.7

The percentage contribution of carbohydrates, proteins and lipids to total energy intake was similar among the different sexes and age groups, as they were among the macro-regions. The averages for the caloric contribution of carbohydrates, proteins and lipids were 54.0%, 15.0%, 31.0% among girls, and 53.0%, 16.0% and 30.0% among boys, respectively (Table 2). Regarding free sugar (added sugar and that present in natural fruit juices), distinct patterns of consumption were observed, with energy contribution from sugar being the greatest among female adolescents, with average values of approximately 25.0% among girls and 22.0% among boys, both of which are above recommended (< 10.0%) by the Brazilian Ministry of Health^15^. Regardless of sex and age, the caloric contribution of saturated fatty acids exceeded the maximum recommended limit of less than 10.0% of total energy intake^15^, with an average of approximately 11.0%.

Micronutrients that had the highest prevalence of inadequacy (> 50.0%) were calcium, phosphorus and vitamins E and A. The inadequacy of vitamin E and calcium consumption was present in 100% of the adolescents, regardless of sex, age or region (Tables 3 and 4). Regarding sodium, more than 80.0% of the adolescents showed an intake higher than the tolerable upper intake level, with 100% of boys presenting even higher figure. Inadequate iron intake was higher among females, especially those in the 14 to 17 years age group (14.0% of girls with inadequate intake against 0.9% of boys). The prevalence of inadequate phosphorus consumption was also higher in girls, reaching 60.0% of adolescents aged 14 to 17 years, while for the boys of the same age group, the inadequacy prevalence was 26.0%. Whereas for vitamin C, the values of inadequacy were higher among male adolescents, being 23.0% for boys aged between 14 and 17 years, and 8.0% for girls in the same age group. Vitamin B12 and zinc were the nutrients with the lowest prevalence of inadequacy. Zinc intake was adequate in 99.0% of male adolescents aged 14 to 17 years as was B12 in 100% of the girls aged between 12 and 13 years. The prevalences of inadequacy were generally similar among the macro-regions.

**Table 3:** Nutritional recommendation (NR)a, percentiles 10, 50 and 90 and prevalences (%IN) of inadequate micronutrient consumption for female adolescents according to age group, both for Brazil and its macroregions. ERICA, 2013-2014.

Micronutrients by macroregions	Age group									

	12-13 years (N = 10,971)					14-17 years (N = 28,941)				
	
	NR	10	50	90	%IN	NR	10	50	90	%IN
Calcium (mg)										
Brazil	1,100	332	519	781	99.0	1,100	344	538	805	99.0
North		329	517	774	99.0		331	519	778	99.0
Northeast		335	522	789	99.0		336	525	781	99.0
Midwest		349	545	812	99.0		351	545	817	99.0
Southeast		340	529	793	99.0		341	533	800	99.0
South		342	537	802	99.0		347	540	808	99.0
Phosphorus (mg)										
Brazil	1,050	709	963	1,284	65.0	1,050	736	997	1,325	60.0
North		734	998	1,329	59.0		734	996	1,326	60.0
Northeast		728	991	1,320	61.0		728	990	1,320	61.0
Midwest		718	976	1,302	63.0		716	975	1,300	63.0
Southeast		723	984	1,313	62.0		723	984	1,313	62.0
South		719	975	1,307	63.0		719	977	1,304	63.0
Iron (mg)										
Brazil	5.7	8.3	11.6	16.0	2.5^b^	7.9	8.6	12.1	16.6	14.1
North		8.4	11.8	16.2	2.1		8.4	11.8	16.2	14.6
Northeast		8.4	11.8	16.2	2.1		8.4	11.9	16.3	14.6
Midwest		8.5	11.9	16.4	2.3		8.5	12.0	16.4	14.6
Southeast		8.5	12.0	16.4	2.1		8.5	11.9	16.4	14.6
South		8.5	12.0	16.5	2.4		8.6	12.0	16.5	14.6
Sodium (mg)										
Brazil	2,200	2,048	2,831	3,820	84.0^c^	2,300	2,154	2,831	3,820	85.0
North		2,220	3,049	4,095	91.0		2,202	3,021	4,064	87.0
Northeast		2,173	2,987	4,014	89.0		2,152	2,957	3,980	85.0
Midwest		2,047	2,819	3,813	84.0		2,028	2,791	3,769	79.0
Southeast		2,128	2,932	3,945	88.0		2,103	2,901	3,397	82.0
South		2,089	2,871	3,876	86.0		2,058	2,840	3,837	80.0
Zinc (mg)										
Brazil	7.0	8.1	11.0	14.7	3.0	7.3	8.3	11.3	15.1	3.0
North		8.1	11.1	14.9	3.0		8.2	11.1	14.9	4.0
Northeast		8.2	11.1	15.0	3.0		8.2	11.1	14.9	4.0
Midwest		8.3	11.3	15.2	2.0		8.3	11.3	15.2	3.0
Southeast		8.2	11.2	15.0	3.0		8.2	11.2	15.0	4.0
South		8.3	11.2	15.0	3.0		8.3	11.3	15.1	4.0
Vitamin A (mg)										
Brazil	420	194	350	624	66.0	485	208	374	665	72.0
North		207	373	664	60.0		207	372	662	72.0
Northeast		205	368	655	61.0		204	368	655	73.0
Midwest		202	364	648	63.0		202	364	647	74.0
Southeast		203	365	649	62.0		201	363	647	74.0
South		201	363	639	63.0		200	360	645	74.0
Vitamin B12 (mcg)										
Brazil	1.5	2.6	4.1	6.3	0.0	2.0	2.7	4.3	6.6	1.0
North		2.7	4.2	6.5	0.0		2.7	4.2	6.5	2.0
Northeast		2.7	4.2	6.5	0.0		2.7	4.2	6.5	2.0
Midwest		2.7	4.2	6.5	0.0		2.7	4.2	6.5	2.0
Southeast		2.7	4.2	6.5	0.0		2.7	4.2	6.5	2.0
South		2.7	4.2	6.5	0.0		2.7	4.2	6.4	2.0
Vitamin E (mg)										
Brazil	9.0	2.8	3.9	5.4	100.0	12.0	2.9	4.0	5.4	100.0
North		2.6	3.6	4.9	100.0		2.6	3.7	5.0	100.0
Northeast		2.7	3.7	5.1	100.0		2.8	3.8	5.2	100.0
Midwest		3.0	4.2	5.6	100.0		3.1	4.3	5.7	100.0
Southeast		2.8	3.9	5.3	100.0		2.9	4.0	5.4	100.0
South		3.0	4.1	5.5	100.0		3.0	4.1	5.6	100.0
Vitamin C (mg)										
Brazil	39	54.7	93.2	155.1	2.0	56	59.2	100.4	167.0	8.0
North		61.2	104.0	172.4	1.0		60.5	103.0	171.3	7.0
Northeast		59.2	100.9	167.8	1.0		58.8	100.0	166.2	8.0
Midwest		56.6	96.6	161.0	2.0		56.2	95.6	159.3	10.0
Southeast		57.2	97.4	162.5	1.0		56.6	96.3	160.9	10.0
South		55.4	94.9	158.0	2.0		55.0	93.9	157.5	11.0

^a^ Nutritional recommendation based on EAR (Estimated Average Requirements).

^b^ Estimated using the probabilistic approach method for which it was possible to estimate the standard error.

^c^ Estimated based on the tolerable upper intake level.

**Table 4:** Nutritional recommendation (NR)a, percentiles 10, 50 and 90 and prevalences (%IN) of inadequate micronutrient consumption for male adolescents according to age group, both for Brazil and its macroregions. ERICA, 2013-2014.

Micronutrients by macroregions	Age group									

	12-13 years (N = 8,983)					14-17 years (N = 23,076)				
	
	NR	10	50	90	%IN	NR	10	50	90	%IN
Calcium (mg)										
Brazil	1,100	333	558	898	97.0	1,100	371	617	982	95.0
North		335	563	728	97.0		341	573	919	96.0
Northeast		344	576	926	96.0		349	586	943	96.0
Midwest		377	629	1,005	94.0		385	638	1,016	93.0
Southeast		353	592	949	96.0		358	599	961	95.0
South		363	608	971	95.0		365	613	979	95.0
Phosphorus (mg)										
Brazil	1,050	808	1,088	1,453	45.0	1,050	909	1,219	1,617	26.0
North		838	1,133	1,516	38.0		844	1,145	1,529	36.0
Northeast		849	1,151	1,537	36.0		859	1,161	1,549	34.0
Midwest		900	1,215	1,620	27.0		911	1,227	1,638	26.0
Southeast		863	1,170	1,560	33.0		871	1,175	1,567	32.0
South		877	1,187	1,576	31.0		885	1,196	1,594	29.0
Iron (mg)										
Brazil	5.9	10.1	13.7	18.1	0.6^b^	7.7	11.6	15.5	20.2	0.9
North		10.3	14.0	18.5	0.6		10.5	14.2	18.8	1.6
Northeast		10.7	14.4	19.0	0.6		10.8	14.6	19.2	1.6
Midwest		11.5	15.5	20.3	0.6		11.7	15.7	20.5	0.9
Southeast		11.0	14.8	19.5	0.6		11.1	15.0	19.7	1.3
South		11.4	15.1	19.9	0.6		11.4	15.3	20.2	0.9
Sodium (mg)										
Brazil	2,200	2,572	3,432	4,506	97.0^c^	2,300	2,880	3.8174	4,995	99.0
North		2,715	3,637	4,802	98.0		2,722	3,649	4,817	98.0
Northeast		2,717	3.651	4,828	99.0		2,736	3,668	4,837	98.0
Midwest		2,788	3,727	4,925	99.0		2,790	3,736	4,921	98.0
Southeast		2,738	3,666	4,832	98.0		2,751	3,680	4,856	98.0
South		2,760	3,688	4,865	99.0		2,764	3,701	4,887	98.0
Zinc (mg)										
Brazil	7.0	9.3	12.8	17.5	1.0	8.5	10.5	14.4	19.5	2.0
North		9.1	12.6	17.3	1.0		9.3	12.9	17.6	5.0
Northeast		9.5	13.1	18.0	1.0		9.7	13.4	18.3	4.0
Midwest		10.9	15.0	20.4	0.0		11.2	15.3	20.9	1.0
Southeast		9.9	13.7	18.6	0.0		10.1	14.0	19.0	3.0
South		10.3	14.2	19.3	0.0		10.6	14.6	19.8	2.0
Vitamin A (mg)										
Brazil	445	217	369	617	68.0	630	238	403	676	87.0
North		238	406	681	59.0		237	405	679	86.0
Northeast		234	398	668	61.0		233	397	665	87.0
Midwest		227	387	652	63.0		226	386	649	89.0
Southeast		229	391	655	62.0		224	382	642	88.0
South		224	386	646	64.0		224	382	642	89.0
Vitamin B12 (mcg)										
Brazil	1.5	2.6	4.4	7.3	1.0	2.0	3.0	5.0	8.3	1.0
North		2.6	4.4	7.3	1.0		2.7	4.5	7.5	3.0
Northeast		2.7	4.6	7.6	0.0		2.8	4.7	7.8	2.0
Midwest		3.2	5.3	8.8	0.0		3.2	5.4	9.0	1.0
Southeast		2.9	4.7	7.8	0.0		2.9	4.8	8.0	2.0
South		2.9	4.9	8.2	0.0		3.0	5.0	8.3	1.0
Vitamin E (mg)										
Brazil	9.0	2.9	4.3	6.2	100.0	12.0	3.2	4.7	6.8	100.0
North		2.7	4.0	5.8	100.0		2.8	4.1	5.9	100.0
Northeast		2.9	4.3	6.1	100.0		3.0	4.4	6.3	100.0
Midwest		3.5	5.0	7.1	99.0		3.6	5.2	7.3	100.0
Southeast		3.1	4.5	6.5	100.0		3.2	4.7	6.7	100.0
South		3.3	4.8	6.9	99.0		3.4	5.0	7.0	100.0
Vitamin C (mg)										
Brazil	39.0	37.2	91.7	210.0	11.0	63.0	42.9	104.6	236.0	23.0
North		42.5	104.8	240.0	8.0		42.5	104.0	237.0	23.0
Northeast		41.4	102.5	233.0	9.0		41.2	101.7	233.0	24.0
Midwest		40.8	101.7	229.6	9.0		41.0	101.1	230.3	24.0
Southeast		39.5	98.5	228.2	10.0		39.6	98.6	225.0	26.0
South		39.6	96.6	221.4	10.0		39.3	96.6	222.1	27.0

^a^ Nutritional recommendation based on EAR (Estimated Average Requirements).

^b^ Estimated using the probabilistic approach method for which it was possible to estimate the standard error.

^c^ Estimated based on the tolerable upper intake level.

## DISCUSSION

Brazilian adolescents’ diets were characterized by the consumption of traditional foods, such as rice and beans, with a high prevalence of sugary drinks, such as juices and carbonated soft drinks, and ultra-processed food intake. This dietary profile was accompanied by an excessive consumption of saturated fatty acids and free sugar, as it was with a high prevalence of inadequate intake of micronutrients such as calcium, vitamins A and E. In addition, more than 80.0% of the adolescents showed a sodium intake above the recommended maximum limits.

Until now, only two national surveys evaluated food consumption by adolescents, both of which were performed by the IBGE: *Pesquisa Nacional de Saúde do Escolar* (PeNSE *–* The Brazilian National Survey of School Health)^f^ and the *Inquérito Nacional de Alimentação* (INA *–* Brazilian National Dietary Survey)^g^. PeNSE was performed in 2009 and 2012 during the ninth year of elementary school and evaluated dietary intake using questions regarding the frequency in which foods, considered as healthy and non-healthy, were consumed. The 2008-2009 INA was a home survey that evaluated food consumption in individuals aged 10 years or more using food records from two non-consecutive days. However, despite the methodological differences used to evaluate dietary intake, the food consumption pattern found during ERICA was similar to that observed among adolescents evaluated in the 2008-2009 INA and during the last PeNSE, in 2012.

In this study, rice, beans, bread, juices and uncarbonated soft drinks, and beef were the foods most consumed by adolescents regardless of sex and age. These foods, with the exception of juices and uncarbonated soft drinks, were also the most consumed among the adolescents involved in the INA^20^. Regarding the consumption of sugary drinks, the prevalence of ingesting juices, uncarbonated soft drinks and sodas during ERICA was greater than what was observed during INA. The consumption of juices and uncarbonated soft drinks was reported by more than 50.0% of the adolescents participating in ERICA, while in INA the prevalence was about 44.0%. In relation to the intake of carbonated soft drinks, the consumption prevalence of these was almost twice as large in ERICA when compared to the INA results (45.0% *versus* 28.0%, respectively). The prevalence of carbonated soft drink intake in adolescents involved in ERICA was also higher than the value observed in the 2012 PeNSE, in which approximately 33.0% of adolescents reported consuming carbonated soft drinks on five or more days during the week.

During ERICA, there were distinct food patterns observed among the different Brazilian macro-regions, which showed a higher prevalence of carbonated soft drink intake in the South region (51.0%). Whereas, vegetable consumption was highest in the Midwest region (54.0%), which was a result similar to that observed during the 2012 PeNSE (51.2%).

The average energy intake observed among adolescents during ERICA was higher compared to the estimated averages for adolescents from the INA. During ERICA, the energy values ranged from 2,036 kcal to 2,124 kcal among female adolescents, and from 2,281 kcal to 2,582 kcal among male adolescents, while in the INA these values ranged from 1,869 to 1,912 kcal among girls and 1,952 kcal to 2,198 kcal among boys^24^. This difference can be explained by the increased consumption of high energy density ultra-processed foods such as carbonated soft drinks, juices, biscuits and fried and baked snacks that was observed between the surveys, as well as the difference methods that were used to estimate food consumption, since during the INA there was an estimated underreporting of energy consumption at an average of 17.0%^14^.

Regarding the contribution percentage of total energy intake for macronutrients, the averages estimated in this study for carbohydrates, proteins and lipids were within the limits established by the Brazilian Ministry of Health^15^. We did not observe any significant variation in caloric macronutrient contribution among the macro-regions. Carbohydrate intake was lower among adolescents involved in ERICA when compared to the results of the INA (57.0% *versus* 54.0%), whereas the caloric contribution of lipids in ERICA was greater than that observed in adolescents from the INA (27.0% *versus* 31.0%).

During this study, the estimated average for the caloric contribution of saturated fatty acids was 11.0%, which is above the upper limit of 10.0% of total energy consumption as recommended by the Brazilian Ministry of Health^15^. This value was slightly larger than what was found during the INA, which was approximately 10.0%. The average caloric contribution of free sugar among adolescents evaluated in ERICA ranged from 25.0% among girls to 22.0% among boys and was two times greater than the value recommended by the Brazilian Ministry of Health, which is less than 10.0% of total caloric intake. This high sugar consumption can be explained by the high consumption prevalence of sweets, desserts and sugary drinks, such as carbonated soft drinks, juices and milk drinks. The values found in this study were similar to those observed during the INA, and the caloric contribution of free sugar was higher among female adolescents evaluated in ERICA (22.0% *versus* 25.0%, respectively). The calorie participation from free sugar was higher in the South region, reaching an average of 27.0% of total caloric consumption among girls aged 12 to 13 years. The South region actually showed the highest prevalence of carbonated soft drink consumption, which is a source of free sugar. International population-based studies have results similar to those observed during ERICA in terms of free sugar consumption^4^ ^,^ ^13^. In Canada, the caloric contribution of added sugar among adolescents was 25.0%, with carbonated soft drinks being the main source of free sugar in their diet^4^.

Regarding micronutrients, calcium and sodium were the minerals that were seen to have the highest prevalence of inadequacy: 99.0% of girls with calcium inadequacy, which is higher than the value found in the INA (around 97.0%). The inadequate calcium intake observed in this study may be partly explained by the persistently low prevalence of dairy product intake among adolescents. In relation to sodium consumption, the prevalence of adolescents with consumption above the maximum recommended level ranged from 84.0% among girls, aged 12 to 13 years, to 99.0% among boys, aged 14 to 17 years, which reflects the high intake of high-sodium-content foods, such as crackers and processed meats, by adolescents. The values of the prevalence of sodium inadequacy were higher in ERICA when compared to values of inadequacy from the INA, whose maximum observed value was 89.0% among boys aged between 14 and 18 years.

The prevalence of iron inadequacy was around six times greater in adolescents aged from 14 to 17 years when compared to adolescents aged from 12 to 13 years. The greater prevalence of inadequate iron intake among older girls was also observed among the adolescents from the INA; however, the prevalence of inadequacy was higher than that observed in this study (24.0% *versus* 14.0%, respectively)^4^.

Vitamins A and E were those which had the highest prevalence of inadequacy, especially vitamin E, whose inadequate consumption was observed in 100% of the adolescents. The values observed in this study for these micronutrients are similar to those found among adolescents involved in the INA. However, the prevalences of vitamin C inadequacy among adolescents from ERICA were lower than those obtained during the INA. For example, the prevalence of inadequacy was 2.0% among girls aged 12 to 13 years in ERICA, while in the INA this prevalence was 33.0% among girls aged 10 to 13 years. These values can be partly explained by differences in age and the higher consumption prevalence of juices and uncarbonated soft drinks among the ERICA adolescents, despite the low rate of fruit consumption.

The World Health Organization (WHO)^h^ also established an EAR for iron and vitamins A and B12. However, when compared to the cut-off points established by the IOM, the differences are most significant for vitamin A and iron. The cut-off points proposed by WHO for vitamin A (330-400 mg) are lower than those established by the IOM. As a result, the prevalence of inadequacy values would be lower if the WHO recommendation had been used. The prevalence of vitamin A inadequacy would be about 40.0% and 30.0% for girls and boys, respectively, if the lower recommended limit of 330 mg of vitamin A were considered as the cut-off point. Regarding iron, the EAR values for older age groups (15 to 17 years) were higher than those established by the IOM (9.0 *versus* 7.9 for girls, and 9.6 *versus* 7.7 for boys), as a result, the higher prevalence of inadequacy values were expected. However, employing the EAR method as a cut-off point is not suitable to evaluate iron inadequacy in women of childbearing age, given that the needs present asymmetric distribution. In this case, the probabilistic approach method must be selected, which was true of the choice made in this study^12^. However, when comparing the EAR values for iron, as proposed by WHO, with the percentiles of normal intake estimated for adolescents to older age group, it was concluded that the prevalence of inadequacy values estimated in this study would probably be similar if the cut points established by WHO were used. The prevalence of iron inadequacy would be approximately 10.0% among girls and around 5.0% among boys (data not presented).

ERICA was the first national school-base survey to use the 24-HDR as a method of evaluating food consumption, which made obtaining estimates of energy and nutrient intake possible, as well as enabling better characterization of the quality of adolescents’ diets. The advantages of this method are its low cost, rapid implementation, and the fact that is does not alter the eating habits of the individual being evaluated^24^. Errors in the 24-HDR are mainly related to the interviewee’s memory, thus applying standardized interviews techniques such as in the Multiple-Pass Method are important for reducing the frequency of under-reported food consumption^5^. During ERICA, a second 24-HDR was performed in a sub-sample of adolescents, which allowed the application of statistical methods to estimate the within-person variability used for correcting the distribution of nutrients and for calculating normal consumption and prevalences of nutrient inadequacy^7^.

During the five-year gap between 2008-2009 (INA) and 2013-2014 (ERICA), there was an observed worsening of nutrient inadequacy, such as in calcium and vitamins A and E, which play an important role for adolescents to achieve proper growth and development. These inadequacies coexist with the high intake of nutrients related to the development of NCD^7^ ^,^ ^24^, in particular regarding the high consumption of sodium, saturated fat and free sugar, which reflect the increased prevalence of ultra-processed food intake, such as carbonated soft drinks, juices, uncarbonated soft drinks and low participation of healthy food markers such as milk and fruits, in the diet of this age group.

Reducing the consumption of ultra-processed foods is one of the recommendations set out in the new *Guia Alimentar para População Brasileira* (Dietary Guidelines for the Brazilian Population)^16^. These foods are associated with excessive calorie consumption and higher risk of obesity^17^ ^,^ ^18^. In fact, between 2008-2009 (INA) and 2013-2014 (ERICA) we observed an increase in total energy consumption and the prevalence of obesity among adolescents, which was almost double (4.9% *versus* 9.0%, respectively). Therefore, the results of this study confirm the importance of recommendations targeted towards reducing ultra-processed food consumption and interventions to promote healthy eating habits in adolescents.

## Footnotes

a Instituto Brasileiro de Geografia e Estatística. Pesquisa de Orçamentos Familiares (POF): antropometria e estado nutricional de crianças, adolescentes e adultos no Brasil, 2008-2009. Rio de Janeiro (RJ): Instituto Brasileiro de Geografia e Estatística; 2010 [cited 2015 Sept 10]. Available from: http://www.ibge.gov.br/home/estatistica/populacao/condicaodevida/pof/2008_2009_encaa/pof_20082009_encaa.pdf

b World Health Organization. Maternal, newborn, child and adolescent health: adolescent development. Geneva: World Health Organization; 2014 [cited 2014 Oct 7]. Available from: http://www.who.int/maternal_child_adolescent/topics/adolescence/dev/en/

c Instituto Brasileiro de Geografia e Estatística. Pesquisa de Orçamentos Familiares (POF): análise da disponibilidade domiciliar de alimentos e do estado nutricional no Brasil; 2002-2003. Rio de Janeiro (RJ): Instituto Brasileiro de Geografia e Estatística; 2004 [cited 2015 Sept 10]. p. 76. Available from: http://biblioteca.ibge.gov.br/visualizacao/livros/liv4472.pdf

d Instituto Brasileiro de Geografia e Estatística. Pesquisa de Orçamentos Familiares (POF), 2008-2009: tabela de composição nutricional dos alimentos consumidos no Brasil. Rio de Janeiro (RJ): Instituto Brasileiro de Geografia e Estatística; 2011 [cited 2015 Sept 10]. Available from: http://biblioteca.ibge.gov.br/visualizacao/livros/liv50002.pdf

e Instituto Brasileiro de Geografia e Estatística. Pesquisa de Orçamentos Familiares (POF), 2008-2009: tabela de composição nutricional dos alimentos consumidos no Brasil. Rio de Janeiro (RJ): Instituto Brasileiro de Geografia e Estatística; 2011 [cited 2015 Sept 10]. Available from: http://biblioteca.ibge.gov.br/visualizacao/livros/liv50000.pdf

f Instituto Brasileiro de Geografia e Estatística. Pesquisa Nacional de Saúde do Escolar (PeNSE): 2012. Rio de Janeiro (RJ): Instituto Brasileiro de Geografia e Estatística; 2013 [cited 2015 Sept 10]. Available from: http://biblioteca.ibge.gov.br/visualizacao/livros/liv64436.pdf

g Instituto Brasileiro de Geografia e Estatística. Pesquisa de Orçamentos Familiares (POF), 2008-2009: análise do consumo alimentar pessoal no Brasil. Rio de Janeiro (RJ): Instituto Brasileiro de Geografia e Estatística; 2010 [cited 2015 Sept 10]. p. 150. Available from: http://biblioteca.ibge.gov.br/visualizacao/livros/liv50063.pdf

h World Health Organization. Vitamin and mineral requirements in human nutrition. 2nd ed. Geneva: 2nd ed.
