# Supplementary material for: ERICA: intake of macro and micronutrients of Brazilian adolescents
Source: Rev Saude Publica. 2016 Feb 2;50(Suppl 1):5s. doi: 10.1590/S01518-8787.2016050006698 (PMC4767036; doi:10.1590/S01518-8787.2016050006698)
Supplement: Translation

# Translation: ERICA: ingestão de macro e micronutrientes em adolescentes brasileiros

**Authors:** Amanda de Moura Souza, Laura Augusta Barufaldi, Gabriela de Azevedo Abreu, Denise Tavares Giannini, Cecília Lacroix de Oliveira, Marize Melo dos Santos, Vanessa Sá Leal, Francisco de Assis Guedes Vasconcelos

**Affiliations:** I Instituto de Estudos em Saúde Coletiva. Universidade Federal do Rio de Janeiro. Rio de Janeiro, RJ, Brasil; II Departamento de Vigilância de Doenças e Agravos Não Transmissíveis e Promoção da Saúde. Secretaria de Vigilância em Saúde. Ministério da Saúde. Brasília, DF, Brasil; III Programa de Pós-Graduação em Saúde Coletiva. Instituto de Medicina Social. Universidade do Estado do Rio de Janeiro. Rio de Janeiro, RJ, Brasil; IV Divisão de Nutrição. Hospital Universitário Pedro Ernesto. Universidade do Estado do Rio de Janeiro. Rio de Janeiro, RJ, Brasil; V Departamento de Nutrição. Universidade do Estado do Rio de Janeiro. Rio de Janeiro, RJ, Brasil; VI Departamento de Nutrição. Núcleo de Estudos em Saúde Pública. Universidade Federal do Piauí. Teresina, PI, Brasil; VII Núcleo de Nutrição. Centro Acadêmico de Vitória. Universidade Federal de Pernambuco. Vitória de Santo Antão, PE, Brasil; VIII Departamento de Nutrição. Centro de Ciências da Saúde. Universidade Federal de Santa Catarina. Florianópolis, SC, Brasil

**Keywords:** Adolescente, Consumo de Alimentos, Micronutrientes, Macronutrientes, Inquéritos sobre Dietas, Estudos Transversais, Inquéritos Nutricionais

## Abstract

**OBJETIVO:**

Descrever o perfil de consumo alimentar e de macronutrientes, e estimar a prevalência de inadequação da ingestão de micronutrientes em adolescentes brasileiros.

**MÉTODOS:**

Foram avaliados dados de 71.791 adolescentes de 12 a 17 anos que participaram do Estudo de Riscos Cardiovasculares em Adolescentes (ERICA), realizado em 2013-2014. O consumo alimentar foi estimado por meio de recordatório alimentar de 24h; em uma subamostra dos adolescentes foi coletado um segundo R24h para a estimativa da variância intrapessoal e o cálculo de ingestão alimentar usual. Estimou-se prevalência de consumo de alimentos ou grupos de alimentos referidos pelos adolescentes. Para ingestão inadequada de sódio considerou-se valores acima do nível de ingestão máxima tolerável. Prevalências de inadequação foram calculadas pelo método da Necessidade Média Estimada (*Estimated Average Requirement* – *EAR)* como ponto de corte. Todas as análises foram estratificadas segundo sexo, faixa etária e macrorregiões do País, e levaram em consideração o peso amostral e a complexidade do desenho do estudo.

**RESULTADOS:**

Os alimentos mais consumidos pelos adolescentes foram arroz, feijão e outras leguminosas, sucos e refrescos, pães, e carne bovina. A ingestão média de energia dos adolescentes variou de 2.036 kcal (meninas de 12 a 13 anos) a 2.582 kcal (meninos de 14 a 17 anos). O consumo de ácidos graxos saturados e de açúcar livre ultrapassaram limites máximos recomendados da ingestão energética total (< 10,0%). Maiores prevalências de inadequação (> 50,0%) foram para cálcio e vitaminas A e E. O consumo de sódio foi acima dos limites máximos recomendados em mais de 80,0% dos adolescentes.

**CONCLUSÕES:**

A dieta dos adolescentes brasileiros é caracterizada pelo consumo de alimentos tradicionais, como arroz e feijão, e ingestão elevada de bebidas açucaradas e alimentos ultraprocessados. Esse padrão associa-se à elevada inadequação da ingestão de cálcio, vitaminas A e E e ao consumo excessivo de ácidos graxos saturados, sódio e açúcar livre.

## INTRODUÇÃO

As doenças crônicas não-transmissíveis (DCNT) são a principal causa de morte em todas as regiões do Brasil^19^ ^.^ O sobrepeso e a obesidade, fatores de risco importantes para a ocorrência dessas doenças, apresentam níveis de prevalência crescentes em todo o mundo e atinge todas as fases da vida, inclusive crianças e adolescentes^9^.

O aumento no consumo de alimentos ultraprocessados, ricos em gordura, açúcar e sal, e o baixo consumo de legumes, verduras e frutas^20^, associados ao menor gasto energético diário, explicam as tendências crescentes de sobrepeso e obesidade^2^ ^,^ ^a^ e alterações metabólicas na população infantil e adolescente. Também contribuem para as deficiências nutricionais características nessa fase da vida, como de ferro, zinco, cálcio, fósforo e vitaminas A, C, E^21^.

A adolescência é uma fase de intensa modificação corporal^b^, e hábitos alimentares inadequados estão associados ao risco elevado de obesidade e outras DNCT^8^, sendo importante o monitoramento do consumo alimentar dos adolescentes brasileiros para a implementação e avaliação de estratégias de intervenção. Diante disso, o objetivo do presente estudo foi descrever o perfil de consumo alimentar e de macronutrientes e estimar a prevalência de inadequação da ingestão de micronutrientes em adolescentes brasileiros.

## MÉTODOS

Foram utilizados os dados do Estudo de Riscos Cardiovasculares em Adolescentes (ERICA) realizado no período de 2013 a 2014. O ERICA é um inquérito nacional de base escolar cujo objetivo foi avaliar a prevalência de fatores de risco cardiovasculares e síndrome metabólica em adolescentes de 12 a 17 anos que frequentavam escolas públicas e privadas localizadas em 124 cidades brasileiras. Informações detalhadas quanto ao processo de amostragem e a coleta de dados foram publicadas previamente^3^ ^,^ ^23^. Resumidamente, o ERICA adotou um plano de amostragem por conglomerado em três estágios. No primeiro estágio, foram selecionadas as escolas com probabilidade proporcional ao tamanho, previamente estratificadas em 32 estratos geográficos (27 capitais e cinco conjuntos com os demais municípios de cada macrorregião). No segundo estágio, foram selecionadas três combinações de turno (manhã e tarde) e ano (um dos três últimos anos do ensino fundamental ou de um dos três anos do ensino médio). No terceiro estágio, foi selecionada uma turma para cada uma das combinações descritas anteriormente^15^. Foram excluídos adolescentes fora da faixa etária de 12 a 17 anos que possuíam algum grau de deficiência que impossibilitassem a avaliação antropométrica e o preenchimento do questionário, e também as adolescentes gestantes.

Dos 102.327 adolescentes elegíveis para participarem do estudo, 73.160 responderam o recordatório alimentar de 24 horas (R24h) e 75.589 preencheram, em coletor eletrônico de dados (*Personal Digital Assistant* – PDA), o questionário do adolescente (com cerca de 100 questões divididas em 11 blocos, abordando aspectos sociodemográficos, de saúde e de estilo de vida). Os participantes do ERICA foram agrupados em subconjuntos de acordo com as partes do estudo para as quais se tinham informação, de forma que os pesos amostrais foram calculados para cada um dos subconjuntos definidos. No presente estudo foram, portanto, avaliados 71.971 adolescentes que tinham dados completos para o subconjunto de PDA e R24h. A taxa de não reposta para este subconjunto foi de 29,7%. Em uma subamostra de dois adolescentes por turma (cerca de 7,0% da amostra), foram coletados um segundo R24h, utilizado para estimativa da variância intrapessoal, o que possibilitou o cálculo ingestão alimentar usual dos adolescentes.

A ingestão alimentar foi estimada pela aplicação de R24h. Os adolescentes foram entrevistados por pesquisadores de campo devidamente treinados, que utilizaram um *software* específico para a entrada de dados de consumo alimentar, com registro direto das informações em *netbooks*. A técnica de entrevista utilizada foi o *multiple-pass method* ^5^, que consiste em uma entrevista orientada em cinco etapas, com o objetivo de reduzir o subrelato do consumo alimentar. O *software* utilizado continha uma lista de alimentos provenientes da base de dados de aquisição de alimentos e bebidas da Pesquisa de Orçamentos Familiares de 2002-2003 realizada pelo Instituto Brasileiro de Geografia e Estatística (IBGE)^c^. Os alimentos que não constavam na base de dados foram incluídos pelos entrevistadores.

A ingestão de energia e nutrientes foi estimada com base na Tabela de Composição Nutricional dos Alimentos Consumidos no Brasil^d^ e na Tabela de Medidas Referidas para os Alimentos Consumidos no Brasil^e^.

Os dados de ingestão de nutrientes não incluíram o consumo de suplementos ou medicamentos. Para análise da ingestão de energia e nutrientes, considerou-se a adição de óleo de soja em todas as formas de preparação cozidas e refogadas de carnes e vegetais. O consumo usual de açúcar e adoçante foi avaliado a partir da seguinte questão: “utiliza com frequência”; com as seguintes opções de resposta: açúcar, adoçante, açúcar e adoçante, não utiliza. Padronizou-se a adição de 10 g de açúcar para cada 100 ml de suco de fruta, café, café com leite, chá e mate, quando os adolescentes reportaram o consumo usual de açúcar; e adição de 5 g de açúcar para cada 100 ml dessas bebidas, quando foi reportado o consumo usual de açúcar e adoçante. Os 1.626 itens alimentares disponíveis na lista de alimentos utilizada no *software* específico para coleta de dados do ERICA foram categorizados em 35 grupos com perfis similares de macronutrientes (Tabela 1). Um único dia de consumo fornece boas estimativas da média populacional da ingestão de nutrientes e alimentos^22^; portanto, as estimativas de prevalência de consumo dos alimentos e das médias populacionais de ingestão de energia e da contribuição percentual de macronutrientes foram calculadas com base em um R24h. Foram apresentados somente os 20 alimentos mais consumidos.

**Tabela 1:** Categorização dos alimentos citados pelos participantes do ERICA segundo perfil similar de macronutrientes. ERICA, Brasil, 2013-2014.

Grupos de alimentos	Descrição
Arroz	Arroz, arroz com legumes, *sushi* e outras preparações à base de arroz
Milho	Milho, farinha de milho, polenta e outras preparações à base de milho
Feijões e outras leguminosas	Feijões, carne de soja e outras leguminosas
Hortaliças	Vegetais folhosos e legumes
Tubérculos	Batatas, exceto industrializadas (*chips*), mandioca, inhame e outros tubérculos
Frutas	Frutas e salada de frutas
Oleaginosas	Amendoim, castanha de caju, amêndoas e outros
Cereais matinais	Aveia, sucrilhos, barra de cereais e outros cereais
Massas	Macarrão, ravióli, lasanha e outras preparações à base de massas
Sopas	Sopas e caldos
Pães	Pães brancos e integrais e torradas
Bolos e tortas	Bolos e tortas em geral
Biscoitos doces	Biscoitos doces e recheados
Biscoitos salgados	Biscoito salgados e tipo *chips* (batata ou milho)
Carne bovina	Carne bovina e preparações à base de carne e outras carnes
Carne de porco	Carne de porco e preparações à base de carne porco
Frango	Frango e preparações à base de frango e outras aves
Peixes	Peixe e preparações à base de peixe
Carnes processadas	Presunto, salame, mortadela, linguiça, salsicha e outras carnes processadas
Ovos	Ovos e preparações a base de ovos
Leite	Leite integral e desnatado
Bebidas lácteas com sabor	Bebidas lácteas adoçadas com aromas artificiais ou naturais, e leite fermentado
Bebidas à base de soja	Leite de soja e bebidas à base de soja
Sucos e refrescos	Sucos de frutas naturais e industrializados
Refrigerantes	Refrigerantes normais
Refrigerantes *diet* ou *light*	Refrigerantes dietéticos e *light*
Café	Café, *cappuccino*, café com leite e outras bebidas à base de café
Chá	Chás
Bebidas alcoólicas	Vinho, cerveja e outros
Queijos e outros produtos lácteos	Queijos e iogurtes
Doces e sobremesas	Doces e sobremesas a base de frutas, chocolate e outras guloseimas
Açúcar, mel e geleias	Açúcar, mel e geleias
Doces e sobremesas *diet* ou *light*	Doces, sobremesas, bolos, tortas e biscoitos doces *diet* ou *light*
Óleos e gorduras	Óleos vegetais, azeite de oliva, manteiga, margarina, molhos e condimentos
Pizza	Pizzas e calzones
Salgados fritos e assados	Coxinha, empadão, pão de queijo e outros salgados
Sanduíches	Hambúrgueres e outros sanduíches

Os percentis das distribuições de ingestão e as prevalências de consumo inadequado de micronutrientes (cálcio, fósforo, ferro, sódio, zinco, vitaminas A, C, E e B12) foram estimados com base nos dados de R24h, corrigidos pela variabilidade intraindividual segundo método proposto pelo *National Cancer Institute* (NCI)^7^. Esse método consiste em um modelo misto não linear de duas partes: a primeira é baseada em um modelo de regressão logística com efeitos aleatórios para estimativa da probabilidade de consumo; a segunda estima a quantidade consumida por modelos de regressão linear com efeitos aleatórios aplicados após a transformação dos dados para normalidade. As cincos macrorregiões do País e a situação da escola (urbano ou rural) foram consideradas como covariáveis em todos os modelos utilizados.

As prevalências de inadequação foram estimadas como a proporção de adolescentes com ingestão do micronutriente inferior à necessidade média estimada^9^ ^-^ ^16^, utilizando o método da Necessidade Média Estimada (*Estimated Average Requirement* – EAR*)* como ponto de corte, conforme recomendado pelo *Institute of Medicine* (IOM)^11^. O cálculo da prevalência de inadequação considerou o peso amostral e a complexidade do desenho da amostra, usando a técnica de replicação *Balanced Repeated Replication* (BRR) com modificação de Fay^1^ ^,^ ^6^.

Para a estimativa da inadequação do ferro, foi utilizada a abordagem probabilística manualmente determinada, uma vez que a curva de distribuição da necessidade de ferro é considerada assimétrica entre as mulheres em idade fértil, não atendendo aos pressupostos necessários para que a EAR seja utilizada^12^. Para cada percentil (1, 5, 10, 15, 25, 40, 50, 75, 85, 90, 95, 99) da distribuição da ingestão usual de ferro foram estimadas probabilidade de inadequação de acordo com o sexo e grupo de idade com base nas recomendações do IOM^12^. A prevalência de inadequação consiste na soma do percentual dos adolescentes com inadequação em cada percentil.

Para ingestão de sódio, valores acima do nível de ingestão máximo tolerável (*Tolerable Upper Intake Level*) foram considerados inadequados, permitindo a estimativa da proporção de adolescentes em risco de efeito adverso à saúde^10^.

A idade foi categorizada em duas faixas etárias devido às diferentes recomendações para ingestão de micronutrientes segundo sexo e idade. As análises foram estratificadas segundo sexo, grupo etário (12 a 13 anos e 14 a 17 anos) e macrorregiões. Todas as estimativas foram calculadas utilizando o *software* SAS (*Statistical Analysis System*) versão 9.3 e levaram em consideração os fatores de expansão e a complexidade do desenho da amostra.

O ERICA foi aprovado pelos Comitês de Ética em Pesquisa do Instituto de Estudos em Saúde Coletiva da Universidade Federal do Rio de Janeiro e de cada estado e do Distrito Federal. Todos os participantes assinaram termo de assentimento.

## RESULTADOS

Os alimentos com maior prevalência de consumo entre os adolescentes foram arroz (82,0%), feijão (68,0%), sucos e refrescos (56,0%), pães (53,0%) e carne bovina (52,0%) nas duas faixas etárias (Figura 1). Observou-se alta prevalência de consumo de alimentos ultraprocessados, como refrigerantes, salgados fritos e assados, e biscoitos doces e salgados, sendo o refrigerante o sexto alimento mais referido (45,0%). A prevalência do consumo de frutas foi baixa, e esse grupo de alimentos ficou entre os 20 mais consumidos somente entre os meninos de 12 a 13 anos (18,0%).

**Figura 1:** Prevalência dos 20 alimentos mais consumidos entre os adolescentes brasileiros segundo sexo e faixa etária. ERICA, 2013-2014.

As análises estratificadas segundo macrorregiões apresentaram diferenças importantes quanto à prevalência de consumo de alguns itens alimentares (Figura 2). O café (64,0%) ficou entre os cinco alimentos mais consumidos somente na região Norte. O feijão foi o segundo alimento mais consumido nas regiões Sudeste, Centro-Oeste e Nordeste. A região Sul apresentou a maior prevalência de consumo de refrigerantes (51,0%). As hortaliças (54,0%) configuraram entre os cinco alimentos mais consumidos somente na região Centro-Oeste.

**Figura 2:** Prevalência dos 20 alimentos mais consumidos entre os adolescentes brasileiros segundo macrorregiões. ERICA, 2013-2014.

A ingestão média de energia dos adolescentes variou de 2.036 kcal entre meninas de 12 a 13 anos a 2.582 kcal entre meninos de 14 a 17 anos (Tabela 2). A ingestão energética total de acordo com a idade não diferiu para o sexo feminino. Para o sexo masculino, a ingestão de energia foi maior entre os meninos de 14 a 17 anos quando comparados aos meninos de 12 a 13 anos (2.582 kcal *versus* 2.281 kcal). As médias de ingestão energética foram similares entre as macrorregiões.

**Tabela 2:** Média e IC95% do consumo de energia e do percentual do consumo calórico total dos macronutrientes segundo sexo e grupo de idade, para o Brasil e macrorregiões. ERICA, 2013-2014.

Variável	Meninas				Meninos			
	
	12-13		14-17		12-13		14-17	
			
	Média	IC95%	Média	IC95%	Média	IC95%	Média	IC95%
Energia (kcal)								
Brasil	2.036	1.981-2.090	2.124	2.086-2.161	2.281	2.192-2.370	2.582	2.511-2.653
Norte	2.121	1.990-2.253	2.155	2.103-2.206	2.261	2.135-2.387	2.573	2.499-2.648
Nordeste	2.100	1.924-2.277	2.257	2.188-2.326	2.502	2.349-2.657	2.668	2.568-2.767
Centro-Oeste	2.068	1.988-2.148	2.112	2.052-2.172	2.394	2.273-2.516	2.652	2.564-2.741
Sudeste	2.018	1.961-2.076	2.085	2.024-2.144	2.213	2.062-2.364	2.564	2.433-2.696
Sul	1.913	1.728-2.098	2.033	1.945-2.121	2.121	1.980-2.261	2.464	2.393-2.536
Carboidratos (%)								
Brasil	54,0	53,4-54,3	53,7	53,4-54,1	53,4	52,9-53,9	53,4	52,8-54,0
Norte	54,8	53,9-55,7	53,8	53,5-54,4	54,7	53,6-55,9	54,8	54,2-55,5
Nordeste	54,6	53,5-55,6	54,0	53,5-54,6	54,4	53,5-55,2	54,6	53,7-55,4
Centro-Oeste	53,2	51,9-54,4	53,0	52,2-53,8	52,1	50,9-53,3	51,5	50,7-52,3
Sudeste	53,5	52,8-54,1	53,5	52,9-54,1	53,1	52,5-53-8	52,9	51,9-53,9
Sul	54,5	53,5-55,5	54,3	52,7-55,8	52,2	49,5-54,9	53,8	52,6-55,0
Proteína (%)								
Brasil	15,4	15,1-15,7	15,4	15,2-15,6	16,2	15,9-16,4	16,3	16,0-16,6
Norte	15,6	15,1-16,1	15,6	15,4-15,9	16,3	15,8-16,9	16,2	15,9-16,5
Nordeste	15,1	14,7-15,4	15,1	14,7-15,6	15,9	15,2-16,6	15,7	15,5-16,0
Centro-Oeste	15,9	15,4-16,5	15,8	15,5-16,1	16,7	16,0-17,4	17,1	16,7-17,5
Sudeste	15,6	15,0-16,1	15,6	15,3-15,9	16,2	15,9-16,6	16,5	16,0-17,1
Sul	14,8	14,0-15,5	14,7	14,1-15,3	16,2	15,3-17,0	15,8	15,4-16,2
Lipídeos totais (%)								
Brasil	30,9	30,5-31,3	31,1	30,7-31,5	30,6	30,2-31,0	30,2	29,8-30,6
Norte	29,5	29,0-30,0	30,6	30,0-31,2	28,8	28,1-29,5	28,7	28,2-29,1
Nordeste	30,6	29,7-31,4	31,0	30,4-31,6	29,8	29,1-30,5	29,6	28,7-30,5
Centro-Oeste	31,3	30,4-32,1	31,5	31,0-32,0	31,4	30,6-32,2	31,5	31,0-32,0
Sudeste	31,3	30,6-32,0	31,2	30,5-31,8	30,9	30,3-31,5	30,4	29,9-31,0
Sul	31,1	30,3-31,9	31,3	30,3-32,3	31,7	29,9-33,6	30,4	29,4-31,4
Ácidos graxos saturados (%)								
Brasil	11,3	11,1-11,7	11,5	11,3-11,7	10,9	10,6-11,1	10,8	10,7-11,0
Norte	10,5	10,1-10,8	11,2	10,8-11,5	10,3	10,0-10,7	10,2	9,9-10,5
Nordeste	11,3	10,8-11,7	11,5	11,1-11,9	10,8	10,3-11,3	10,8	10,3-11,3
Centro-Oeste	11,5	10,9-12,2	11,4	11,1-11,7	11,1	10,7-11,5	10,9	10,7-11,2
Sudeste	11,6	11,1-12,0	11,5	11,2-11,9	10,9	10,6-11,2	10,9	10,7-11,1
Sul	11,2	10,7-11,6	11,4	11,0-11,9	11,0	10,3-11,8	10,9	10,5-11,3
Ácidos graxos monoinsaturados (%)								
Brasil	10,5	10,3-10,7	10,6	10,4-10,7	10,3	10,0-10,5	10,2	10,0-10,6
Norte	10,3	10,0-10,6	10,3	10,2-10,5	10,0	9,7-10,2	9,9	9,7-10,0
Nordeste	10,8	10,6-11,0	11,0	10,8-11,1	10,4	10,2-10,6	10,4	10,3-10,5
Centro-Oeste	10,9	10,5-11,2	10,9	10,7-11,1	10,6	10,4-10,9	10,8	10,6-11,0
Sudeste	10,3	10,1-10,5	10,4	10,3-10,6	10,3	10,8-10,4	10,1	10,0-10,3
Sul	10,9	10,5-11,2	10,7	10,5-10,9	10,6	10,3-10,9	10,7	10,5-10,9
Ácidos graxos poli-insaturados (%)								
Brasil	6,2	6,1-6,3	6,1	6,0-6,2	6,2	5,9-6,5	5,9	5,8-6,0
Norte	6,0	5,7-6,3	5,9	5,7-6,0	5,4	5,1-5,7	5,4	5,3-5,6
Nordeste	5,7	5,4-5,9	5,8	5,6-6,0	5,5	5,3-5,7	5,4	5,3-5,5
Centro-Oeste	5,9	5,7-6,2	6,2	6,0-6,3	6,1	5,8-6,4	6,1	6,0-6,3
Sudeste	6,4	6,2-6,6	6,2	6,0-6,4	6,7	6,1-7,2	6,1	5,9-6,3
Sul	6,5	6,1-6,9	6,4	6,1-6,7	6,6	6,3-6,9	6,2	5,9-6,5
Ácidos graxos trans total (%)								
Brasil	1,0	0,9-1,0	1,0	0,9-1,0	1,0	0,9-1,0	1,0	0,9-1,0
Norte	1,1	1,0-1,2	1,1	1,0-1,2	1,1	1,0-1,1	1,1	1,0-1,2
Nordeste	1,0	0,9-1,1	1,0	0,9-1,0	1,0	0,9-1,2	1,0	1,0-1,1
Centro-Oeste	0,9	0,9-0,9	0,9	0,9-1,0	1,0	1,0-1,1	1,0	0,9-1,0
Sudeste	0,9	0,8-1,0	0,9	0,9-1,0	1,0	0,8-1,1	1,0	0,9-1,1
Sul	1,3	1,1-1,5	1,2	1,1-1,2	1,3	1,2-1,4	1,3	1,2-1,4
Açúcar total (%)								
Brasil	24,7	24,1-25,3	24,7	24,2-25,1	21,8	21,0-22,5	21,9	21,4-22,4
Norte	21,3	19,6-22,9	21,5	20,9-22,1	19,2	18,0-20,4	19,1	18,4-19,7
Nordeste	24,1	23,3-24,9	24,7	23,7-25,6	22,3	21,3-23,4	22,0	21,3-22,8
Centro-Oeste	26,5	25,5-27,6	25,5	24,9-26,1	22,8	21,7-23,9	22,4	21,6-23,2
Sudeste	24,7	23,7-25,7	24,7	24,1-25,3	21,6	20,3-22,9	21,8	21,0-22,7
Sul	27,0	26,0-28,1	26,3	24,8-27,7	22,6	20,6-24,5	23,9	23,2-24,7

A contribuição percentual de carboidratos, proteínas e lipídeos para a ingestão energética total foi semelhante nos diferentes grupos de sexo e idade, bem como entre as macrorregiões. As médias de contribuição calórica de carboidratos, proteínas e lipídios foram de 54,0%, 15,0%, 31,0% entre as meninas e de 53,0%, 16,0% e 30,0% entre os meninos, respectivamente (Tabela 2). Para o açúcar livre (açúcar de adição e o presente nos sucos de frutas naturais), foram observados padrões distintos de consumo, sendo a contribuição energética proveniente do açúcar maior entre adolescentes do sexo feminino, com valores médios de aproximadamente 25,0% entre as meninas e 22,0% entre os meninos, ambos acima do recomendado (< 10,0%) pelo Ministério da Saúde^15^. Independentemente do sexo e da idade, a contribuição calórica dos ácidos graxos saturados ultrapassou o limite máximo recomendado de menos de 10,0% da ingestão energética total^15^, com média de aproximadamente 11,0%.

Os micronutrientes que apresentaram as maiores prevalências de inadequação (> 50,0%) foram cálcio, fósforo e vitaminas E e A. A inadequação do consumo de vitamina E e cálcio atingiram 100% dos adolescentes, independentemente de sexo, idade e região (Tabelas 3 e 4). Em relação ao sódio, mais de 80,0% dos adolescentes apresentaram ingestão superior ao valor de ingestão máximo tolerável, sendo que para os meninos esse valor foi de quase 100%. A inadequação do consumo de ferro foi maior entre o sexo feminino, especialmente na faixa etária de 14 a 17 anos (14,0% das meninas com inadequação contra 0,9% dos meninos). A prevalência de inadequação do consumo de fósforo também foi maior nas meninas, atingindo 60,0% das adolescentes de 14 a 17 anos, ao passo que nos meninos da mesma faixa etária a prevalência de inadequação foi de 26,0%. Já para vitamina C, os valores de inadequação foram maiores entre os adolescentes do sexo masculino, sendo de 23,0% para os meninos de 14 a 17 anos e 8,0% para as meninas da mesma faixa etária. A vitamina B12 e o zinco foram os nutrientes com as menores prevalências de inadequação. A ingestão de zinco foi adequada para 99,0% dos adolescentes do sexo masculino de 14 a 17 anos e a de B12 foi adequada para 100% das meninas de 12 a 13 anos. As prevalências de inadequação foram em geral similares entre as macrorregiões.

**Tabela 3:** Recomendação nutricional (RN)a, percentis 10, 50 e 90 e prevalências (%IN) de inadequação do consumo de micronutrientes para adolescentes do sexo feminino segundo faixa etária, para o Brasil e macrorregiões. ERICA, 2013-2014.

Micronutrientes por macrorregiões	Faixa etária									

	12-13 anos (N = 10.971)					14-17 anos (N = 28.941)				
	
	RN	10	50	90	%IN	RN	10	50	90	%IN
Cálcio (mg)										
Brasil	1.100	332	519	781	99,0	1.100	344	538	805	99,0
Norte		329	517	774	99,0		331	519	778	99,0
Nordeste		335	522	789	99,0		336	525	781	99,0
Centro-Oeste		349	545	812	99,0		351	545	817	99,0
Sudeste		340	529	793	99,0		341	533	800	99,0
Sul		342	537	802	99,0		347	540	808	99,0
Fósforo (mg)										
Brasil	1.050	709	963	1.284	65,0	1.050	736	997	1.325	60,0
Norte		734	998	1.329	59,0		734	996	1.326	60,0
Nordeste		728	991	1.320	61,0		728	990	1.320	61,0
Centro-Oeste		718	976	1.302	63,0		716	975	1.300	63,0
Sudeste		723	984	1.313	62,0		723	984	1.313	62,0
Sul		719	975	1.307	63,0		719	977	1.304	63,0
Ferro (mg)										
Brasil	5,7	8,3	11,6	16,0	2,5^b^	7,9	8,6	12,1	16,6	14,1
Norte		8,4	11,8	16,2	2,1		8,4	11,8	16,2	14,6
Nordeste		8,4	11,8	16,2	2,1		8,4	11,9	16,3	14,6
Centro-Oeste		8,5	11,9	16,4	2,3		8,5	12,0	16,4	14,6
Sudeste		8,5	12,0	16,4	2,1		8,5	11,9	16,4	14,6
Sul		8,5	12,0	16,5	2,4		8,6	12,0	16,5	14,6
Sódio (mg)										
Brasil	2.200	2.048	2.831	3.820	84,0^c^	2.300	2.154	2.831	3.820	85,0
Norte		2.220	3.049	4.095	91,0		2.202	3.021	4.064	87,0
Nordeste		2.173	2.987	4.014	89,0		2.152	2.957	3.980	85,0
Centro-Oeste		2.047	2.819	3.813	84,0		2.028	2.791	3.769	79,0
Sudeste		2.128	2.932	3.945	88,0		2.103	2.901	3.397	82,0
Sul		2.089	2.871	3.876	86,0		2.058	2.840	3.837	80,0
Zinco (mg)										
Brasil	7,0	8,1	11,0	14,7	3,0	7,3	8,3	11,3	15,1	3,0
Norte		8,1	11,1	14,9	3,0		8,2	11,1	14,9	4,0
Nordeste		8,2	11,1	15,0	3,0		8,2	11,1	14,9	4,0
Centro-Oeste		8,3	11,3	15,2	2,0		8,3	11,3	15,2	3,0
Sudeste		8,2	11,2	15,0	3,0		8,2	11,2	15,0	4,0
Sul		8,3	11,2	15,0	3,0		8,3	11,3	15,1	4,0
Vitamina A (mg)										
Brasil	420	194	350	624	66,0	485	208	374	665	72,0
Norte		207	373	664	60,0		207	372	662	72,0
Nordeste		205	368	655	61,0		204	368	655	73,0
Centro-Oeste		202	364	648	63,0		202	364	647	74,0
Sudeste		203	365	649	62,0		201	363	647	74,0
Sul		201	363	639	63,0		200	360	645	74,0
Vitamina B12 (mcg)										
Brasil	1,5	2,6	4,1	6,3	0,0	2,0	2,7	4,3	6,6	1,0
Norte		2,7	4,2	6,5	0,0		2,7	4,2	6,5	2,0
Nordeste		2,7	4,2	6,5	0,0		2,7	4,2	6,5	2,0
Centro-Oeste		2,7	4,2	6,5	0,0		2,7	4,2	6,5	2,0
Sudeste		2,7	4,2	6,5	0,0		2,7	4,2	6,5	2,0
Sul		2,7	4,2	6,5	0,0		2,7	4,2	6,4	2,0
Vitamina E (mg)										
Brasil	9,0	2,8	3,9	5,4	100,0	12,0	2,9	4,0	5,4	100,0
Norte		2,6	3,6	4,9	100,0		2,6	3,7	5,0	100,0
Nordeste		2,7	3,7	5,1	100,0		2,8	3,8	5,2	100,0
Centro-Oeste		3,0	4,2	5,6	100,0		3,1	4,3	5,7	100,0
Sudeste		2,8	3,9	5,3	100,0		2,9	4,0	5,4	100,0
Sul		3,0	4,1	5,5	100,0		3,0	4,1	5,6	100,0
Vitamina C (mg)										
Brasil	39	54,7	93,2	155,1	2,0	56	59,2	100,4	167,0	8,0
Norte		61,2	104,0	172,4	1,0		60,5	103,0	171,3	7,0
Nordeste		59,2	100,9	167,8	1,0		58,8	100,0	166,2	8,0
Centro-Oeste		56,6	96,6	161,0	2,0		56,2	95,6	159,3	10,0
Sudeste		57,2	97,4	162,5	1,0		56,6	96,3	160,9	10,0
Sul		55,4	94,9	158,0	2,0		55,0	93,9	157,5	11,0

^a^ Recomendação nutricional com base na EAR (*Estimated Average Requirements*).

^b^ Estimado pelo método da abordagem probabilística para qual não foi possível estimar o erro-padrão.

^c^ Estimado com base no nível de ingestão máxima tolerável.

**Tabela 4:** Recomendação nutricional (RN)a, percentis 10, 50 e 90 e prevalências (%IN) de inadequação do consumo de micronutrientes para adolescentes do sexo masculino segundo faixa etária, para o Brasil e macrorregiões. ERICA, 2013-2014.

Micronutrientes por macrorregiões	Faixa etária									

	12-13 anos (N = 8.983)					14-17 anos (N = 23.076)				
	
	RN	10	50	90	%IN	RN	10	50	90	%IN
Cálcio (mg)										
Brasil	1.100	333	558	898	97,0	1.100	371	617	982	95,0
Norte		335	563	728	97,0		341	573	919	96,0
Nordeste		344	576	926	96,0		349	586	943	96,0
Centro-Oeste		377	629	1005	94,0		385	638	1.016	93,0
Sudeste		353	592	949	96,0		358	599	961	95,0
Sul		363	608	971	95,0		365	613	979	95,0
Fósforo (mg)										
Brasil	1.050	808	1.088	1.453	45,0	1.050	909	1.219	1.617	26,0
Norte		838	1.133	1.516	38,0		844	1.145	1.529	36,0
Nordeste		849	1.151	1.537	36,0		859	1.161	1.549	34,0
Centro-Oeste		900	1.215	1.620	27,0		911	1.227	1.638	26,0
Sudeste		863	1.170	1.560	33,0		871	1.175	1.567	32,0
Sul		877	1.187	1.576	31,0		885	1.196	1.594	29,0
Ferro (mg)										
Brasil	5,9	10,1	13,7	18,1	0,6^b^	7,7	11,6	15,5	20,2	0,9
Norte		10,3	14,0	18,5	0,6		10,5	14,2	18,8	1,6
Nordeste		10,7	14,4	19,0	0,6		10,8	14,6	19,2	1,6
Centro-Oeste		11,5	15,5	20,3	0,6		11,7	15,7	20,5	0,9
Sudeste		11,0	14,8	19,5	0,6		11,1	15,0	19,7	1,3
Sul		11,4	15,1	19,9	0,6		11,4	15,3	20,2	0,9
Sódio (mg)										
Brasil	2.200	2.572	3.432	4.506	97,0^c^	2.300	2.880	3.8174	4.995	99,0
Norte		2.715	3.637	4.802	98,0		2.722	3.649	4.817	98,0
Nordeste		2.717	3.651	4.828	99,0		2.736	3.668	4.837	98,0
Centro-Oeste		2.788	3.727	4.925	99,0		2.790	3.736	4.921	98,0
Sudeste		2.738	3.666	4.832	98,0		2.751	3.680	4.856	98,0
Sul		2.760	3.688	4.865	99,0		2.764	3.701	4.887	98,0
Zinco (mg)										
Brasil	7,0	9,3	12,8	17,5	1,0	8,5	10,5	14,4	19,5	2,0
Norte		9,1	12,6	17,3	1,0		9,3	12,9	17,6	5,0
Nordeste		9,5	13,1	18,0	1,0		9,7	13,4	18,3	4,0
Centro-Oeste		10,9	15,0	20,4	0,0		11,2	15,3	20,9	1,0
Sudeste		9,9	13,7	18,6	0,0		10,1	14,0	19,0	3,0
Sul		10,3	14,2	19,3	0,0		10,6	14,6	19,8	2,0
Vitamina A (mg)										
Brasil	445	217	369	617	68,0	630	238	403	676	87,0
Norte		238	406	681	59,0		237	405	679	86,0
Nordeste		234	398	668	61,0		233	397	665	87,0
Centro-Oeste		227	387	652	63,0		226	386	649	89,0
Sudeste		229	391	655	62,0		224	382	642	88,0
Sul		224	386	646	64,0		224	382	642	89,0
Vitamina B12 (mcg)										
Brasil	1,5	2,6	4,4	7,3	1,0	2,0	3,0	5,0	8,3	1,0
Norte		2,6	4,4	7,3	1,0		2,7	4,5	7,5	3,0
Nordeste		2,7	4,6	7,6	0,0		2,8	4,7	7,8	2,0
Centro-Oeste		3,2	5,3	8,8	0,0		3,2	5,4	9,0	1,0
Sudeste		2,9	4,7	7,8	0,0		2,9	4,8	8,0	2,0
Sul		2,9	4,9	8,2	0,0		3,0	5,0	8,3	1,0
Vitamine E (mg)										
Brasil	9,0	2,9	4,3	6,2	100,0	12,0	3,2	4,7	6,8	100,0
Norte		2,7	4,0	5,8	100,0		2,8	4,1	5,9	100,0
Nordeste		2,9	4,3	6,1	100,0		3,0	4,4	6,3	100,0
Centro-Oeste		3,5	5,0	7,1	99,0		3,6	5,2	7,3	100,0
Sudeste		3,1	4,5	6,5	100,0		3,2	4,7	6,7	100,0
Sul		3,3	4,8	6,9	99,0		3,4	5,0	7,0	100,0
Vitamina C (mg)										
Brasil	39,0	37,2	91,7	210,0	11,0	63,0	42,9	104,6	236,0	23,0
Norte		42,5	104,8	240,0	8,0		42,5	104,0	237,0	23,0
Nordeste		41,4	102,5	233,0	9,0		41,2	101,7	233,0	24,0
Centro-Oeste		40,8	101,0	229,6	9,0		41,0	101,1	230,3	24,0
Sudeste		39,5	98,5	228,2	10,0		39,6	98,6	225,0	26,0
Sul		39,6	96,6	221,4	10,0		39,3	96,6	222,1	27,0

^a^ Recomendação nutricional com base na EAR (*Estimated Average Requirements*).

^b^ Estimado pelo método da abordagem probabilística para qual não foi possível estimar o erro-padrão.

^c^ Estimado com base no nível de ingestão máxima tolerável.

## DISCUSSÃO

A dieta dos adolescentes brasileiros caracterizou-se pela manutenção do consumo de alimentos tradicionais, como arroz e feijão, e elevada prevalência de consumo de bebidas açucaradas, como sucos e refrigerantes, e de alimentos ultraprocessados. Esse perfil dietético acompanhou o consumo excessivo de ácidos graxos saturados e açúcar livre, bem como a elevada prevalência de inadequação da ingestão de micronutrientes como cálcio, vitaminas A e E. Além disso, mais de 80,0% dos adolescentes apresentaram o consumo de sódio acima dos limites máximos recomendados.

Até o presente momento, somente dois inquéritos nacionais, ambos conduzidos pelo IBGE, avaliaram o consumo alimentar em adolescentes: a Pesquisa Nacional de Saúde do Escolar (PeNSE)^f^ e o Inquérito Nacional de Alimentação (INA)^g^. A PeNSE foi realizada nos anos de 2009 e 2012 em escolares do nono ano do ensino fundamental e avaliou a ingestão alimentar utilizando questões relativas à frequência de consumo de alimentos considerados marcadores de alimentação saudável e não saudável. O INA 2008-2009 foi um inquérito domiciliar que avaliou o consumo alimentar em indivíduos com 10 anos ou mais com base em dois registros alimentares. Contudo, apesar das diferenças metodológicas na avaliação da ingestão dietética, o padrão de consumo alimentar encontrado no ERICA foi similar ao observado entre os adolescentes avaliados no INA 2008-2009 e na última PeNSE de 2012.

No presente estudo, arroz, feijão, pães, sucos e refrescos e carne bovina foram os alimentos mais consumidos pelos adolescentes independentemente do sexo e faixa etária. Esses alimentos, exceto sucos e refrescos, também foram os mais consumidos entre os adolescentes do INA^20^. Em relação ao consumo de bebidas açucaradas, a prevalência de ingestão de sucos, refrescos e refrigerantes no ERICA foi maior do que a observada no INA. O consumo de sucos e refrescos foi reportado por mais de 50,0% dos adolescentes participantes do ERICA, ao passo que no INA a prevalência foi de cerca de 44,0%. Em relação à ingestão de refrigerantes, a prevalência de consumo foi quase duas vezes maior no ERICA quando comparado aos resultados do INA (45,0% *versus* 28,0%, respectivamente). A prevalência de ingestão de refrigerantes nos adolescentes do ERICA também foi superior ao valor observado na PeNSE 2012, onde aproximadamente 33,0% dos adolescentes reportaram a ingestão de refrigerantes em cinco dias ou mais da semana.

No ERICA, padrões alimentares distintos foram observados entre as macrorregiões brasileiras, com destaque para a maior prevalência de ingestão de refrigerantes na região Sul (51,0%). Já o consumo de hortaliças foi maior na região Centro-Oeste (54,0%), resultado similar ao observado na PeNSE 2012 (51,2%).

A ingestão média de energia observada entre os adolescentes do ERICA foi maior quando comparada às médias estimadas para os adolescentes do INA. No ERICA, os valores de energia variaram de 2.036 kcal a 2.124 kcal entre adolescentes do sexo feminino e de 2.281 kcal a 2.582 kcal entre adolescentes do sexo masculino, ao passo que no INA esses valores variaram de 1.869 kcal a 1.912 kcal entre as meninas e de 1.952 kcal a 2.198 kcal entre os meninos^24^. Essa diferença pode ser explicada pelo aumento do consumo de alimentos ultraprocessados de alta densidade energética como refrigerantes, sucos, biscoitos e salgados fritos e assados observados entre os inquéritos, bem como pela diferença dos métodos utilizados para a estimativa do consumo alimentar, já que no INA a estimativa de subrelato do consumo energético foi em média de 17,0%^14^.

Quanto à contribuição percentual dos macronutrientes para a ingestão energética total, as médias estimadas no presente estudo para carboidratos, proteínas e lipídios estão dentro dos limites estabelecidos pelo Ministério da Saúde^15^. Não houve variação importante na contribuição calórica de macronutrientes entre as macrorregiões. Quando comparado aos resultados do INA, o consumo de carboidrato foi menor entre os adolescentes do ERICA (57,0% *versus* 54,0%), ao passo que a contribuição calórica dos lipídeos foi maior do que a observada nos adolescentes do INA (27,0% *versus* 31,0%).

No presente estudo, a média estimada para a contribuição calórica dos ácidos graxos saturados foi 11,0% e está acima do limite máximo de 10,0% do consumo energético total recomendado pelo Ministério da Saúde^15^. Esse valor foi ligeiramente superior ao encontrado no INA, que foi de aproximadamente de 10,0%. A média de contribuição calórica do açúcar livre entre os adolescentes avaliados no ERICA variou de 25,0% entre as meninas a 22,0% entre os meninos e foi duas vezes maior que o valor recomendado pelo Ministério da Saúde de menos de 10,0% do consumo calórico total. O consumo elevado de açúcar pode ser explicado pela alta prevalência de consumo de doces, sobremesas e bebidas açucaradas, como refrigerantes, sucos e bebidas lácteas. Os valores encontrados no presente estudo são similares aos observados no INA, sendo que a contribuição calórica do açúcar livre foi maior entre as adolescentes do sexo feminino avaliadas no ERICA (22,0% *versus* 25,0%, respectivamente). A participação calórica do açúcar livre foi maior na região Sul, atingindo a média de 27,0% do consumo calórico total entre as meninas de 12 a 13 anos. De fato, a região Sul apresentou a maior prevalência de consumo de refrigerantes, fonte de açúcar livre. Estudos internacionais de base populacional apresentam resultados semelhantes aos observados no ERICA quanto ao consumo de açúcar livre^4^ ^,^ ^13^. No Canadá, a contribuição calórica do açúcar de adição entre os adolescentes foi de 25,0%, sendo que o refrigerante foi a principal fonte de açúcar livre da dieta^4^.

Em relação aos micronutrientes, cálcio e sódio foram os minerais que apresentaram as maiores prevalências de inadequação: 99,0% das meninas com inadequação de cálcio, valor superior ao encontrado no INA (cerca de 97,0%). A inadequação do consumo de cálcio observada no presente estudo pode ser explicada, em parte, pela persistência da baixa prevalência de ingestão de leite e derivados entre os adolescentes. Quanto ao consumo de sódio, a prevalência de adolescentes com o consumo acima do nível máximo recomendado variou de 84,0% entre as meninas de 12 a 13 anos a 99,0% entre os meninos de 14 a 17 anos, refletindo a elevada ingestão de alimentos com alto teor de sódio pelos adolescentes, como biscoitos salgados e carnes processadas. Os valores de prevalência de inadequação de sódio foram maiores no ERICA quando comparados aos valores de inadequação obtidos no INA, cujo valor máximo observado foi de 89,0% entre os meninos de 14 a 18 anos.

Para o ferro, a prevalência de inadequação foi cerca de seis vezes maior nas adolescentes de 14 a 17 anos quando comparadas às adolescentes de 12 a 13 anos. Maior prevalência de inadequação do consumo de ferro entre meninas mais velhas também foi observada entre as adolescentes do INA; porém, a prevalência de inadequação foi maior que a observada no presente estudo (24,0% *versus* 14,0%, respectivamente)^4^.

As vitaminas A e E foram as que apresentaram maior prevalência de inadequação, com destaque para a vitamina E, cujo consumo inadequado atingiu 100% dos adolescentes. Os valores encontrados no presente estudo para esses micronutrientes são similares aos encontrados entre os adolescentes do INA. No entanto, as prevalências de inadequação de vitamina C entre os adolescentes do ERICA foram menores do que as obtidas no INA. Por exemplo, entre as meninas de 12 a 13 anos a prevalência de inadequação foi de 2,0% no ERICA, ao passo que no INA esta prevalência foi de 33,0% entre as meninas de 10 a 13 anos. Esses valores podem ser explicados em parte pelas diferenças na faixa etária e pela maior prevalência de consumo de sucos e refrescos entre os adolescentes do ERICA, apesar da manutenção do baixo consumo de frutas.

A Organização Mundial da Saúde (OMS)^h^ também estabelece EAR para ferro e vitaminas A e B12. Entretanto, quando comparados aos pontos de corte estabelecidos pelo IOM, as diferenças mais relevantes são para a vitamina A e o ferro. Os pontos de cortes propostos pela OMS para vitamina A (330-400 mg) são menores do que os estabelecidos pelo IOM; consequentemente, seriam encontrados valores de prevalência de inadequação mais baixos caso fosse utilizada a recomendação da OMS. A prevalência de inadequação de vitamina A seria de cerca de 40,0% e 30,0% para meninas e meninos, respectivamente, se considerado como ponto de corte o limite inferior da recomendação de vitamina A de 330 mg. Para o ferro, os valores de EAR para as faixas etárias mais velhas (15 a 17 anos) são superiores aos estabelecidos pelo IOM (9,0 *versus* 7,9 para meninas e 9,6 *versus* 7,7 para meninos), como consequência, maiores valores de prevalência de inadequação são esperados. No entanto, o método da EAR como ponto de corte não pode ser utilizado para avaliação da inadequação de ferro em mulheres em idade fértil, visto que as necessidades apresentam distribuição assimétrica. Nesse caso, deve-se optar pelo método da abordagem probabilística, o qual foi utilizado no presente estudo^12^. Contudo, ao compararmos os valores de EAR para o ferro propostos pela OMS com os percentis de consumo usual estimados para os adolescentes de faixa etária mais velha, conclui-se que provavelmente os valores de prevalência de inadequação estimados no presente estudo seriam semelhantes caso fossem utilizados os pontos de corte estabelecidos pela OMS. A prevalência de inadequação de ferro seria de aproximadamente 10,0% entre as meninas e cerca de 5,0% entre os meninos (dados não apresentados).

O ERICA é o primeiro inquérito nacional de base escolar que utilizou como método de avaliação do consumo alimentar o R24h, possibilitando a obtenção de estimativas da ingestão energética e de nutrientes, além de melhor caracterização da qualidade da dieta dos adolescentes. Esse método tem como vantagens o seu baixo custo, a aplicação rápida, e a não alteração do hábito alimentar do indivíduo avaliado^25^. Os erros do R24h estão relacionados principalmente à memória do entrevistado, por isso a aplicação de técnicas de entrevistas padronizadas como o *Multiple-Pass Method* são importantes para a redução do sub-relato do consumo alimentar^5^. No ERICA, um segundo R24h foi realizado em uma subamostra dos adolescentes, o que permitiu a aplicação de métodos estatísticos para a estimativa da variabilidade intraindividual utilizada para a correção da distribuição dos nutrientes e para o cálculo do consumo usual e das prevalências de inadequação de nutrientes^7^.

Nos cinco anos decorridos de 2008-2009 (INA) a 2013-2014 (ERICA) foi observado agravamento da inadequação de nutrientes como cálcio e vitaminas A e E, que desempenham papel importante para o crescimento e desenvolvimento adequado do adolescente. Essas inadequações coexistem com a elevada ingestão de nutrientes relacionados ao desenvolvimento de DCNT^20^ ^,^ ^24^, em especial o alto consumo de sódio, gordura saturada e açúcar livre, que refletem o aumento da prevalência de ingestão de alimentos ultraprocessados como refrigerantes, sucos, refrescos e biscoitos e a baixa participação de alimentos marcadores de alimentação saudável como leite e frutas na dieta deste grupo etário.

A redução do consumo de alimentos ultraprocessados é uma das recomendações do novo Guia Alimentar para População Brasileira^16^. Esses alimentos estão associados ao consumo excessivo de calorias e maior de risco de obesidade^17^ ^,^ ^18^. De fato, entre os anos 2008-2009 (INA) e 2013-2014 (ERICA), foram observados o aumento do consumo energético total e da prevalência de obesidade, quase duas vezes, entre os adolescentes (4,9% *versus* 9,0%, respectivamente). Portanto, os resultados do presente estudo reafirmam a importância de recomendações direcionadas para redução do consumo de alimentos ultraprocessados e de intervenções para promoção de hábitos alimentares saudáveis em adolescentes.

## Footnotes

a Instituto Brasileiro de Geografia e Estatística. Pesquisa de Orçamentos Familiares (POF): antropometria e estado nutricional de crianças, adolescentes e adultos no Brasil, 2008-2009. Rio de Janeiro (RJ): Instituto Brasileiro de Geografia e Estatística; 2010 [citado 2015 set 10]. Disponível em: http://www.ibge.gov.br/home/estatistica/populacao/condicaodevida/pof/2008_2009_encaa/pof_20082009_encaa.pdf

b World Health Organization. Maternal, newborn, child and adolescent health: adolescent development. Geneva: World Health Organization; 2014 [citado 2014 out 7]. Disponível em: http://www.who.int/maternal_child_adolescent/topics/adolescence/dev/en/

c Instituto Brasileiro de Geografia e Estatística. Pesquisa de Orçamentos Familiares (POF): análise da disponibilidade domiciliar de alimentos e do estado nutricional no Brasil; 2002-2003. Rio de Janeiro (RJ): Instituto Brasileiro de Geografia e Estatística; 2004 [citado 2015 set 10]. p. 76. Disponível em: http://biblioteca.ibge.gov.br/visualizacao/livros/liv4472.pdf

d Instituto Brasileiro de Geografia e Estatística. Pesquisa de Orçamentos Familiares (POF), 2008-2009: tabela de composição nutricional dos alimentos consumidos no Brasil. Rio de Janeiro (RJ): Instituto Brasileiro de Geografia e Estatística; 2011 [citado 2015 set 10]. Disponível em: http://biblioteca.ibge.gov.br/visualizacao/livros/liv50002.pdf

e Instituto Brasileiro de Geografia e Estatística. Pesquisa de Orçamentos Familiares (POF), 2008-2009: tabela de medidas referidas para os alimentos consumidos no Brasil. Rio de Janeiro (RJ): Instituto Brasileiro de Geografia e Estatística; 2011 [citado 2015 set 10]. Disponível em: http://biblioteca.ibge.gov.br/visualizacao/livros/liv50000.pdf

f Instituto Brasileiro de Geografia e Estatística. Pesquisa Nacional de Saúde do Escolar (PeNSE): 2012 [citado 2015 set 10]. Rio de Janeiro (RJ): Instituto Brasileiro de Geografia e Estatística; 2013. Disponível em: http://biblioteca.ibge.gov.br/visualizacao/livros/liv64436.pdf

g Instituto Brasileiro de Geografia e Estatística. Pesquisa de Orçamentos Familiares (POF), 2008-2009: análise do consumo alimentar pessoal no Brasil. Rio de Janeiro (RJ): Instituto Brasileiro de Geografia e Estatística; 2010 [citado 2015 set 10]. p. 150. Disponível em: http://biblioteca.ibge.gov.br/visualizacao/livros/liv50063.pdf

h World Health Organization. Vitamin and mineral requirements in human nutrition. 2nd ed. Geneva: World Health Organization; 2004.
